# The Clinical Course of COVID-19 Disease in a US Hospital System: a Multi-state Analysis

**DOI:** 10.1093/aje/kwaa286

**Published:** 2020-12-22

**Authors:** Aaloke Mody, Patrick G Lyons, Cristina Vazquez Guillamet, Andrew Michelson, Sean Yu, Angella Sandra Namwase, Pratik Sinha, William G Powderly, Keith Woeltje, Elvin H Geng

**Affiliations:** 1 Division of Infectious Diseases, Department of Medicine, Washington University School of Medicine, St. Louis, Missouri, USA; 2 Division of Pulmonary and Critical Care Medicine, Department of Medicine, Washington University School of Medicine, St. Louis, Missouri, USA; 3 Institute for Informatics, Washington University School of Medicine, St. Louis, Missouri, USA; 4 Division of Critical Care, Department of Anesthesiology, Washington University School of Medicine, St. Louis, Missouri, USA; 5 Center for Clinical Excellence, BJC HealthCare, St. Louis, Missouri, USA

**Keywords:** multi-state analysis, longitudinal trajectory, clinical course, COVID-19 hospitalizations, ICU, mechanical ventilation, age-stratified mortality

## Abstract

There is limited data on longitudinal outcomes for COVID-19 hospitalizations that account for transitions between clinical states over time. Using electronic health record data from a St. Louis-region hospital network, we performed multi-state analyses to examine longitudinal transitions and outcomes among hospitalized adults with laboratory-confirmed COVID-19 with respect to fifteen mutually-exclusive clinical states. Between March 15 and July 25, 2020, 1,577 patients were hospitalized with COVID-19 (49.9% male, median age 63 years [IQR 50, 75], 58.8% Black). Overall, 34.1% (95% confidence interval [CI] 26.4%, 41.8%) had an ICU admission and 12.3% (CI 8.5%, 16.1%) received invasive mechanical ventilation (IMV). The risk of decompensation peaked immediately after admission, discharges peaked around day 3 to 5, and deaths plateaued between days 7 and 16. At 28 days, 12.6% (CI 9.6%, 15.6%) of patients had died (4.2% [CI 3.2%, 5.2%] received IMV) and 80.8% (CI 75.4%, 86.1%) were discharged. Among those receiving IMV, 39.1% (CI 32.0%, 46.2%) remained intubated after 14 days; after 28 days, 37.6% (CI 30.4%, 44.7%) had died and only 37.7% (CI 30.6%, 44.7%) were discharged. Multi-state methods offer granular characterizations of the clinical course of COVID-19 and provide essential information for guiding both clinical decision-making and public health planning.

Abbreviations:

ICU: intensive care unit

ED: emergency department

NIV: noninvasive ventilation

IQR: interquartile range

CI: confidence interval aHR: adjusted hazard ratio

ORIGINAL UNEDITED MANUSCRIPT

A careful characterization of the clinical course of COVID-19 during hospitalization will offer important insights into patients' prognosis and the anticipated burden and duration of resources required for their care—basic clinical information which is still coming into focus for this novel pathogen. Hospitalized patients may take numerous pathways: some only require brief stays while others deteriorate and require admission to the intensive care unit (ICU) with or without invasive mechanical ventilation (IMV) (1-6). Even if these patients survive, many will experience protracted hospital courses prior to discharge. Deaths could occur immediately after admission or after decompensations later on in the hospitalization. An understanding of how patients transition through multiple clinical states over the course of their hospitalization—and the timing of these transitions—will offer situational awareness and information for clinical decision-making and public health planning as the epidemic continues to evolve.

To date, published data on the hospital course for COVID-19 do not yet provide a comprehensive descriptive picture indicative of the experience in the US. For example, while case series do describe the number or incidence of deaths (1-6), such analyses have not captured movement between multiple clinical states over the course of hospitalization. Additionally, the rapidly evolving nature of the pandemic means that in many reports a substantial proportion patients are still in the midst of their illness (7). These analyses have either presented cross-sectional estimates that do not account for this unequal follow-up time, or have excluded patients with incomplete follow-up time, potentially creating bias in both scenarios (1-9). Furthermore, much of the early data on hospitalizations focus only on critically-ill patients and comes from single-center studies earlier in the epidemic, largely from the worst-hit areas such as Wuhan, China (1-3), Lombardy, Italy (4), and New York City (5, 6) where outcomes may not be representative of outcomes elsewhere. Thus, more rigorous data from regions where the burden of COVID-19 did not exceed the capacity of healthcare systems is needed to inform COVID-19 planning in the US going forward.

To address these needs, we used data from the BJC HealthCare Hospital system in St. Louis and the surrounding regions to examine the totality of experience across a number of clinical

ORIGINAL UNEDITED MANUSCRIPT

conditions (e.g., inpatient floor admission, ICU, death, discharge) in a cohort of patients who were admitted with COVID-19. We used multi-state methods to estimate the proportion of patients in various clinical conditions over time as well as the time spent in and rates of transition from each state. This analytic technique permits a more comprehensive examination of the cascade of outcomes (10) during COVID-19 hospitalizations for informing planning and policy.

## METHODS

*Study population and setting.* We analyzed a cohort of consecutive adult patients with confirmed COVID-19 who were admitted to the BJC Hospital system in the St. Louis region between March 15, 2020 and July 25, 2020. We included all patients aged 18 years or older who were admitted to an inpatient service and either had a positive PCR test for severe acute respiratory syndrome coronavirus 2 (SARS-CoV-2) during admission or within the past 7 days, or who had confirmed COVID-19 disease as an encounter diagnosis. BJC HealthCare is a is a non-profit health system that consists of 15 hospitals—ranging from a 1200-bed academic referral center to a 40-bed rural community hospital—in the St. Louis, southern Illinois, and mid-Missouri regions. It serves a diverse population across the range of socioeconomic and sociodemographic spectrums in both urban and rural regions with a catchment area of approximately 3 million peoples (11). During the COVID-19 epidemic, BJC hospitals opened up additional ICUs to manage patients with COVID-19, but never exceeded health care capacity with regards to hospital beds, ICU beds, mechanical ventilators, or staffing. General hospital management protocols are detailed in Web Appendix.

*Measurements.* We extracted data for this analysis from the electronic health record (EHR) for the entire BJC system (Epic; Verona, WI). Data included admission and discharge dates, sociodemographic information, laboratory results, COVID-19 diagnosis codes, flowsheets, level of care (i.e. inpatient floor, ICU), procedures (i.e., intubation), and outcomes (i.e., death, discharge) for all patients as it was charted throughout their hospitalization in the EHR. As part of BJC's routine and

ORIGINAL UNEDITED MANUSCRIPT

ongoing COVID-19 tracking efforts, all patients admitted with COVID-19 had their chart manually reviewed to determine whether they were a resident of a long-term care facility. Additionally, we performed targeted chart reviews (n=27) to reconcile potential inconsistencies in COVID-diagnoses, level of care, and outcomes from the EHR data.

*Analyses.* We sought to assess the clinical course of patients with COVID-19 presenting to the hospital in a manner that accounted for the numerous changes in clinical status patients may have over their hospitalization (e.g., admission, critical illness, intubation, death, discharge) (12-14). We first categorized patients into one of fifteen mutually exclusive and exhaustive states based on their clinical status at each time point: (1) emergency department [ED], (2) inpatient floor, (3) ICU admission without IMV, (4) noninvasive ventilation [NIV], (5) IMV in ICU, (6) NIV after IMV, (7) ICU after IMV, (8) inpatient floor after ICU admission without IMV, (9) inpatient floor after IMV, (10) discharged without ICU admission, (11) discharged with history of ICU admission without IMV, (12) discharged with history of IMV, (13) died, (14) died with history of ICU admission without IMV, and (15) died with history of IMV (Figure 1). We then examined outcomes longitudinally in several ways to highlight unique aspects of patients' clinical courses.

First, we applied nonparametric multi-state analytic techniques based on the Aalen-Johansen method to account for patient movements into and out of multiple clinical states over time and where the observation time for each patient is unequal (12-15). We estimated the probability over time of a patient having a particular clinical status after entering into one of three different states: 1) after inpatient admission, 2) after ICU admission, 3) after NIV, and 4) after endotracheal intubation. For each analysis, time zero was the entry into that particular clinical state and patients were censored at the time of discharge, death, or the end of the observation period (i.e., July 25, 2020).

Second, we estimated the instantaneous rate for ICU admission, NIV, intubation, death, and discharge after inpatient admission (regardless of movements through intermediate states) in order to characterize transition dynamics between clinical states. Additionally, we also estimated transition

ORIGINAL UNEDITED MANUSCRIPT

intensities (i.e., instantaneous rate of transition to the next immediate state) after entering the inpatient floor (state 2), ICU (state 3), NIV (state 4), or IMV (state 5) states (Figure 1).

Third, we used an alluvial diagram to depict the trajectories of individual patients through clinical states over their hospitalization, stratifying by patients' outcome at 28 days. This analysis was restricted to patients with at least 28 days of observation (including time after death or discharge).Fourth, we estimated the duration of the overall hospitalization, ICU admissions, NIV, and IMV based on results from the multi-state analyses.

Fifth, we assessed the cumulative incidence of ICU admission, NIV, intubation, and death by 28 days since inpatient admission stratifying by patient subgroups. We also performed Cox proportional-hazards models to identify patient characteristics independently associated with the time from inpatient admission to ICU admission, intubation, and death. We selected covariates using directed acyclic graphs based on a priori hypotheses of causal relationships between baseline sociodemographic and clinical characteristics and patient outcomes. We examined the proportional hazards assumption using Schoenfeld residuals (16).

Lastly, to assess the changes in patient outcomes over time and explore the potential impact of the introduction of evidence-based therapies (i.e., remdesivir (17) and dexamethasone (18) in moderate or severe disease), we obtained adjusted age-stratified estimates of patient outcomes based on the time period in which they were admitted (i.e., March 15 to May 3 [prior to remdesivir availability] or May 4 to July 25 [after remdesivir availability]). We report these as marginal estimates of age-stratified Poisson models adjusted for sex, race, comorbidities, and whether the patient lived in a long-term care facility.

All analyses were conducted using R 3.2.4 using the *mstate* package (13, 14) and Stata MP 16.1.

RESULTS

ORIGINAL UNEDITED MANUSCRIPT

*Patient characteristics.* Between March 15 and July 25, 2020, 2,940 patients who presented to the ED were confirmed to have COVID-19, and 1,577 were admitted to the hospital (Web Figure 1). Among those hospitalized, 571 were subsequently admitted to the ICU, 343 received NIV, and 213 patients received IMV (Table 1). Median age was 63 years old (IQR 50, 75) and 927 patients (58.8%) were Black (Table 1). As the pandemic progressed, patients admitted later on were younger, had fewer comorbidities, were less likely to be Black, and were less likely to reside in a long-term care facility. They were more likely to be treated with remdesivir and steroids and less likely to be treated with tocilizumab and hydroxychloroquine (Web Table 1). Overall, Black patients tended to be younger, less likely to be male, and to have more comorbidities (Web Table 2).

*Clinical course of COVID-19 hospitalizations based on multi-state analyses.* Overall, 34.1% (95% confidence interval [CI]: 26.4%, 41.8%) of hospitalized patients were in the ICU at some point during admission (including patients receiving IMV) and 12.3% (95% CI: 8.5%, 16.1%) received IMV (Figures 2 and 3, Web Table 3). After admission, the rate of transfer to the ICU and intubation peaked on hospital day 1 and declined thereafter, whereas the rate of discharge peaked between hospital day 3 to 5, and the rate of death plateaued on days 7 through 16 (Figure 4). At 7 days, 51.6% (95% CI: 47.5%, 55.6%) of patients had been discharged and 5.7% (95% CI: 3.7%, 7.7%) had died. At 28 days, 80.8% (95% CI: 75.4%, 86.1%; 20.2% [95% CI: 17.4%, 23%] with a history of ICU admission and 4.3% [95% CI: 3.3%, 5.3%] with a history of IMV) of patients had been discharged and 12.6% (95% CI: 9.6%, 15.6%; 8.6% [95% CI: 6.6%, 10.6%] with an ICU admission and 4.2% [95% CI: 3.2%, 5.2%] with IMV) had died (Figure 2, Web Table 3). The median duration for all inpatient admissions was 5.7 days (IQR 2.9, 11.9). Median duration was 4.2 days (IQR 2.1, 7.4) for those only on the wards, 8.1 days (IQR 4.3, 15.4) for those admitted to the ICU without receiving NIV or IMV, 14.1 days (IQR 7.3, 25.8) for who received NIV but no IMV, and 19.1 days (IQR 10.1, 30.7) for those who received IMV (Figure 5, Web Table 5).

ORIGINAL UNEDITED MANUSCRIPT

Among patients admitted to the ICU and those who received NIV, 50.8% (95% CI: 35%, 66.6%) and 39.5% (95% CI: 26.6%, 52.4%) received IMV at some point, respectively (Figure 2, Web Table 3). The rate of noninvasive and invasive ventilation peaked immediately after ICU transfer; whereas the rate of death (without intubation) peaked around day 5, and the rate of transfer to the ward peaked at day 3 and again at day 12 (Figure 6). At 7 days after ICU admission, 53.9% (95% CI: 40%, 67.8%) remained in the ICU (13.6% [95% CI: 9.4%, 17.7%] receiving NIV and 29.3% [95% CI: 23.8%, 34.8%] receiving IMV), 17.4% (95% CI: 11.5%, 23.2%) were discharged from the hospital and 14.3% (95% CI: 8.7%, 19.9%) had died (6.8% [95% CI: 4%, 9.7%] after IMV). At 28 days, 11.2% (95% CI: 5.2%, 17.2%) remained in the ICU (6.5% [95% CI: 4.1%, 9.0%] receiving IMV), 52.9% (95% CI: 42.4%, 63.4%) were discharged (18.4% [95% CI: 14.0%, 22.8%] had received IMV), and 30.3% (95% CI: 22.5%, 38%) had died (18.0% [95% CI: 13.5%, 22.4%] after IMV) (Figure 2, Web Table 3). The median duration of ICU admissions was 1.9 days (IQR 1.1%, 3.2 days) without NIV or IMV, 4.5 days (IQR 2.0, 9.2) with NIV only, and 10.3 days (IQR 4.6, 20.1) for those who received IMV (Figure 5, Web Table 5).

Lastly, among patients who received IMV, the rate of extubation increased through day 14, while the hazard for death plateaued between days 5 and 12 (Figure 6). At 14 days after intubation, 35.1% (95% CI: 28.2%, 42%) remained on IMV and 28.0% (95% CI: 21.1%, 35%) had died. At 28 days, 16.2% (95% CI: 8.2%, 24.3%) remained in the ICU (10.8% [95% CI: 6.7%, 14.8%] still receiving IMV), 37.6% (95% CI: 30.4%, 44.7%) had died, and only 37.7% (95% CI: 30.6%, 44.7%) had been discharged (Figure 2, Web Table 3). The median duration of IMV was 7.2 days (IQR 2.9, 14.2) (Figure 5, Web Table 4).

In stratified multi-state and multivariable Cox proportional hazards analyses, older patients had markedly increased mortality (adjusted hazard ratio [aHR] 7.00, 95% CI: 2.97, 16.5) for patients greater than 70 years compared to those less than 50), and trended towards increased ICU admissions and receiving NIV and IMV. Long-term care residents also had increased mortality (aHR 1.89, 95% CI: 1.40, 2.54). Men were more likely to be admitted to the ICU (aHR 1.53, 95% CI: 1.29,

ORIGINAL UNEDITED MANUSCRIPT

1.81), receive NIV (aHR 1.34, 95% CI: 1.08, 1.66), and IMV (aHR 1.54, 95% CI: 1.16, 2.02), and potentially trended towards increased mortality. Patients with comorbidities trended towards increased mortality in stratified but not multivariable analyses. Race was not significantly associated with ICU admissions, NIV, IMV, nor death. Lastly, being admitted between May 4 and July 25 (as opposed to earlier in the pandemic) was not associated with changes in rates of ICU admissions, NIV, or IMV but was associated with decreased mortality (aHR 0.66, 95% CI 0.48, 0.91) (Figure 7, Table 2, Web Tables 5 and 6). Decreases in mortality appeared greatest in older patients (Figure 8, Web Table 7).

ORIGINAL UNEDITED MANUSCRIPT

DISCUSSION

We used multi-state analytic methods to longitudinally characterize the clinical course of COVID-19 disease after presentation to the hospital in a manner that accounts for patient transitions between multiple clinical states over the course of admission and the timing of these transitions. We found that at 7 days after hospital admission, 51.6% of patients had been discharged and 5.7% had died; at 28 days, 80.8% had been discharged (20.2% were admitted to the ICU and 4.3% received IMV) and 12.6% had died (8.6% with an ICU admission and 4.2% received IMV). The risk of decompensation was greatest immediately after admission, discharges peaked around day 3 to 5, and mortality plateaued between days 7 and 16. Among patients receiving IMV, 35.1% remained intubated and 28.0% had died after 14 days. Overall, these findings provide a more nuanced and comprehensive depiction of the trajectories of COVID-19 disease after presentation to the hospital.

Our study provides granular epidemiologic data on the clinical course of COVID-19 that are both essential for guiding public health officials in assessing their health systems' capacity and also immediately relevant for clinical decision-making (19). Early in the epidemic, one the primary concerns was the anticipated strain that unmitigated SARS-CoV2 spread was expected to place on health systems, and several influential disease models sought to specifically asses health systems capacity in terms of hospital beds and mechanical ventilators (20, 21). Our analysis details what happens to patients after being hospitalized with COVID-19—including during different phases of the pandemic—and can guide health systems in appropriately planning for the healthcare resources that may be required. In particular, detailed data on the time spent in various clinical states can parameterize disease models to better project needs for staffing, hospital beds, critical care beds, and mechanical ventilators (22-24). Additionally, it offers healthcare providers a complete depiction of the trajectory patients are likely to take based on a patients' current clinical state and the probability of being in other clinical states at different time points further into hospitalization (e.g., patients receive IMV for only a median of 7 days, but 28 days after intubation only 37.7% were discharged, 37.6% had died, and 24.7% remained hospitalized). This level of granularity provides both public health officials and clinicians valuable insights for guiding public health responses as well as making the most informed care decisions with patients and their families.

Our study is the first to our knowledge to longitudinally characterize COVID-19 hospitalization trajectories in a way that comprehensively captures patient transitions between clinical cares states over time. Patients frequently transition between the inpatient floor, ICU, and IMV— often more than once during a hospitalization—prior to ultimately being discharged alive or dying. To date, several studies have described COVID-19 hospitalizations (1, 3-6, 25), but most focused only on critically-ill patients and provided cross-sectional estimates that only included patients with known outcomes and excluded patients who may have had prolonged hospitalizations and were still hospitalized (7). Studies that did include censored observations only considered time to single outcome (i.e., in-hospital death) (5), but did not consider intermediate events such as ICU transfers or intubation. Additionally, these estimates did not account for competing events (15), such as hospital discharge, that would preclude the occurrence of an in-hospital mortality event, potentially also contributing to bias (8, 9). This study adds to this existing literature in several ways. We use rigorous longitudinal methods to estimate the incidence and timing of events in the setting where both competing events are present and where the observation time between participants is not equal (8, 9). Additionally, we use these multi-state methods to assess transitions between multiple clinical states—as opposed to a

ORIGINAL UNEDITED MANUSCRIPT

single one—over the course of one's hospitalization (13, 14). Furthermore, most early reports were single-center studies conducted in regions that were the hardest hit by COVID-19, potentially limiting the generalizability of their experiences. In contrast, our data includes a diverse and representative population from a variety of settings (e.g., both academic and community hospitals, rural and urban settings, diverse patients from both affluent and marginalized communities) and across different phases of the pandemic (i.e., before and after the introduction of evidence-based therapies). Thus, it provides one the most comprehensive characterizations of the clinical course of COVID-19 hospitalizations to date.

Our results offer an additional layer of nuance to characterizations of COVID-19-related hospitalizations, but are also consistent with what has been previously reported (1-6, 26, 27). The majority of patients were admitted to the wards and discharged within 3 to 5 days, but an important subset present critically ill (or decompensate early in their hospitalization) and generally experience a protracted hospital course, often with prolonged periods of IMV and a high-risk for mortality. In our cohort, older age was most strongly associated with poor outcomes such as need for IMV and mortality, followed by male sex. Additionally, we found that patients admitted after May 4th (i.e., after remdesivir was introduced in our hospital network) had reduced mortality rates, though patients admitted during this period were also substantially younger and healthier. Still, this association remained even after adjusting for age and comorbidities and may thus also be indicative—though not definitively so—of the positive impact of routine use of these evidence-based therapies for COVID-19 (i.e., remdesivir (17) and dexamethasone (18)), particularly in older patients. Third, Black patients comprised a greater proportion of those admitted with COVID-19 disease, but, once hospitalized, there were no significant differences in outcomes in adjusted models. This is in line with prior studies and likely explained by the systemic disparities that have led to higher risks of acquiring COVID-19 in Black communities (27-32), but limited differences in the actual pathophysiology once one becomes infected. Fourth, there were trends towards increased mortality with additional comorbidities in stratified analyses, but this was not consistent in multivariable regression. Lastly, though outcomes

ORIGINAL UNEDITED MANUSCRIPT

estimates are also similar to those for influenza-associated and general ARDS (33, 34), more work is needed to understand how COVID-19 clinical phenotypes relate to their underlying pathophysiology and how they differ from other disease states (35-38). Ultimately, further research extending these findings is needed to help us understand to whom, when, and what types of interventions and treatments are needed for optimizing our response to COVID-19, both at the individual patient and public health levels.

There are several limitations to this study. First, we leveraged observational EHR data, which may have misclassified some patient outcomes, COVID-19 diagnoses, hospital events, or their timing. In particular, we did not have granular data on patients' disease severity (e.g., oxygenation levels), the exact timing of multiples events occurring within an hour of each other, or the history or circumstances leading up to admission at a BJC hospital (e.g., duration of symptoms, prior events if patient transferred from a different hospital). Second, we obtained adjusted age-stratified outcome estimates by time period prior to explore the potential impact of routine use of evidence-based COVID-19 therapies, but these analyses were not adjusted presenting disease severity and it is still possible that these estimates are affected by residual confounding. Third, our study only included hospitals from a large health system affiliated with an academic medical center where health care capacity was not exceeding and may not necessarily be reflective of outcomes in other regions of the country or the world, particularly places that experienced a COVID-19 epidemic surge that exceeded their health systems' capacity. Still, we did include patients from several hospitals ranging from an academic, quaternary-care medical center to smaller community hospitals located in both urban and rural settings.

In conclusion, we used multi-state analytic methods to provided nuanced characterizations of the clinical course of COVID-19 hospitalizations. Multi-state approaches provide granular descriptions of patients' trajectories over time and offer useful insights on COVID-19 disease for frontline clinicians, disease modelers, up to health system and public health officials.

ORIGINAL UNEDITED MANUSCRIPT

Acknowledgements: We want to thank the Institute for Informatics (I2) at Washington University in St. Louis for assistance with data extraction from the electronic health records.

**Table 1. Baseline Patient Characteristics, BJC Hospital system, St. Louis, MO, 2020 (n=1,577)**

| Patient Characteristic | Inpatient (n=1577) | | | ICU (n=571) | | | NIV (n=343) | | | Intubated (n=213) | | |
|---|---|---|---|---|---|---|---|---|---|---|---|---|
| | Number[a] | Percent | Median (IQR) | Number[a] | Percent | Median (IQR) | Number[a] | Percent | Median (IQR) | Number[a] | Percent | Median (IQR) |
| Male Sex, n (%) | 787 | 49.9 | | 330 | 57.8 | | 198 | 57.7 | | 131 | 61.2 | |
| Median Age, years (IQR) | 1577 | | 63 (50, 75) | 571 | | 65 (54, 76) | 343 | | 66 (56, 75) | 213 | | 65 (55, 74) |
| Race, n (%) | | | | | | | | | | | | |
| Black or African American | 927 | 58.8 | | 327 | 57.3 | | 188 | 54.8 | | 126 | 58.9 | |
| White | 571 | 36.2 | | 210 | 36.8 | | 138 | 40.2 | | 73 | 34.1 | |
| Other | 30 | 1.9 | | 21 | 3.7 | | 8 | 2.3 | | 9 | 4.2 | |
| Unknown | 49 | 3.1 | | 13 | 2.3 | | 9 | 2.6 | | 6 | 2.8 | |
| Long-Term Care Facility, n (%) | 361 | 22.9 | | 151 | 26.4 | | 92 | 26.8 | | 53 | 24.8 | |
| Academic Hospital, n (%) | 662 | 42.0 | | 287 | 50.3 | | 144 | 42.0 | | 132 | 61.7 | |
| Comorbidities | | | | | | | | | | | | |
| Diabetes, n (%) | 677 | 42.9 | | 259 | 45.4 | | 159 | 46.4 | | 95 | 44.4 | |
| Hypertension, n (%) | 1190 | 75.5 | | 436 | 76.4 | | 272 | 79.3 | | 164 | 76.6 | |
| Chronic Kidney Disease, n (%) | 488 | 30.9 | | 185 | 32.4 | | 111 | 32.4 | | 75 | 35.0 | |
| Cardiac Disease, n (%) | 687 | 43.6 | | 265 | 46.4 | | 170 | 49.6 | | 97 | 45.3 | |
| Pulmonary Disease, n (%) | 481 | 30.5 | | 184 | 32.2 | | 113 | 32.9 | | 60 | 28.0 | |
| Tobacco use, n (%) | 626 | 39.7 | | 229 | 40.1 | | 133 | 38.8 | | 74 | 34.6 | |
| Obesity, n (%) | 849 | 53.8 | | 300 | 52.5 | | 188 | 54.8 | | 103 | 48.1 | |
| Baseline Laboratory Values[b] | | | | | | | | | | | | |
| Median hemoglobin level, g/dl (IQR) | 1547 | | 12.5 (11.0, 13.8) | 554 | | 12.0 (10.2, 13.4) | 324 | | 11.6 (9.7, 13.2) | 212 | | 11.1 (9.6, 13.0) |
| Median platelet count, $10^3/mm^3$ (IQR) | 1547 | | 208 (162, 275) | 554 | | 202 (153, 265) | 324 | | 210 (160, 274) | 212 | | 210 (153, 270) |
| Median white blood cell count, $10^3/mm^3$ (IQR) | 1547 | | 7.0 (5.2, 9.8) | 554 | | 8.0 (5.7, 11.2) | 324 | | 8.0 (5.8, 10.9) | 212 | | 10.1 (6.8, 14.6) |
| Median neutrophil count, $10^3/mm^3$ (IQR) | 1519 | | 5.0 (3.4, 7.6) | 529 | | 6.0 (4.0, 9.2) | 300 | | 6.2 (4.1, 9.2) | 193 | | 7.8 (5.4, 11.2) |
| Median lymphocyte | 1519 | | 1.0 | 529 | | 0.9 | 300 | | 1.0 | 193 | | 0.9 |

ORIGINAL UNEDITED MANUSCRIPT

| | | | | | | | | | | | | |
|---|---|---|---|---|---|---|---|---|---|---|---|---|
| count, $10^3/mm^3$(IQR) | | | (0.8, 1.6) | | | (0.7, 1.3) | | | (0.7, 1.3) | | | (0.6, 1.4) |
| Median creatinine level, mg/dL (IQR) | 1527 | | 1.1 (0.8, 1.6) | 547 | | 1.2 (0.8, 1.9) | 320 | | 1.1 (0.8, 1.7) | 205 | | 1.3 (0.9, 2.1) |
| Median aspartate aminotransferase, units/L (IQR) | 1365 | | 44 (30, 67) | 498 | | 54 (35, 81) | 282 | | 56 (39, 85) | 187 | | 65 (45, 113) |
| Median alanine aminotransferase, units/L (IQR) | 1358 | | 28 (18, 45) | 496 | | 30 (20, 50) | 280 | | 32 (21, 56) | 188 | | 35 (23, 65) |
| Median C-reactive protein, mg/L (IQR) | 716 | | 84 (31, 162) | 332 | | 124 (64, 203) | 198 | | 137 (81, 207) | 123 | | 160 (85, 249) |
| Median ferritin, ng/mL (IQR) | 636 | | 597 (280, 1253) | 300 | | 762 (388, 1707) | 178 | | 800 (454, 1593) | 119 | | 1131 (424, 2096) |
| Median D-dimer, ng/mL (IQR) | 698 | | 1075 (653, 2070) | 327 | | 1370 (744, 3403) | 195 | | 1200 (692, 2510) | 117 | | 1861 (995, 5078) |
| <u>Treatments</u> | | | | | | | | | | | | |
| Remdesivir, n (%) | 207 | 13.1 | | 112 | 19.6 | | 90 | 26.2 | | 44 | 20.6 | |
| Steroids[c], n (%) | 408 | 25.9 | | 238 | 41.7 | | 172 | 50.1 | | 115 | 53.7 | |
| Tocilizumab, n (%) | 32 | 2.0 | | 29 | 5.1 | | 22 | 6.4 | | 28 | 13.1 | |
| Hydroxychloroquine, n (%) | 281 | 17.7 | | 150 | 26.3 | | 103 | 30.0 | | 73 | 34.1 | |
| Time Period, n (%) | | | | | | | | | | | | |
| March 15 – May 3 | 786 | 49.8 | | 302 | 52.9 | | 182 | 53.1 | | 122 | 57.0 | |
| May 4 – July 25 | 791 | 50.2 | | 269 | 47.1 | | 161 | 46.9 | | 92 | 43.0 | |

Abbreviations: ICU=intensive-care unit; NIV=noninvasive ventilation

Footnote: [a]Numbers that do not equal the total N indicates presence of missing values. [b]Baseline laboratory values were only included if they were 48 hours of either inpatient admission, ICU admission, or intubation, respectively. [c]Steroid equivalent to dexamethasone 6mg per day.

ORIGINAL UNEDITED MANUSCRIPT

**Table 2 – Cox Proportional Hazards Regression of Factors Associated with ICU Admission, NIV, Intubation, and Death, BJC Hospital system, St. Louis, MO, 2020 (n=1,577)**

| | **ICU Admission** | | | **NIV** | | | **Intubation** | | | **Death** | | |
|---|---|---|---|---|---|---|---|---|---|---|---|---|
| | Adjusted HR | 95% CI | p-value | Adjusted HR | 95% CI | p-value | Adjusted HR | 95% CI | p-value | Adjusted HR | 95% CI | p-value |
| Male Sex | 1.53 | 1.29, 1.81 | <0.001 | 1.34 | 1.08, 1.66 | 0.007 | 1.54 | 1.16, 2.02 | 0.002 | 1.20 | 0.91, 1.59 | 0.20 |
| Age | | | | | | | | | | | | |
| <50 years old | 1.00 | REF | | 1.00 | REF | | 1.00 | REF | | 1.00 | REF | |
| 50-70 years old | 1.48 | 1.16, 1.89 | 0.006 | 1.62 | 1.17, 2.24 | 0.010 | 1.81 | 1.20, 2.74 | 0.012 | 2.91 | 1.23, 6.85 | <0.001 |
| >70 years old | 1.31 | 0.99, 1.73 | | 1.33 | 0.91, 1.92 | | 1.40 | 0.87, 2.25 | | 7.00 | 2.97, 16.48 | |
| Race | | | | | | | | | | | | |
| Black | 1.00 | REF | | 1.00 | REF | | 1.00 | REF | | 1.00 | REF | |
| White | 1.01 | 0.84, 1.21 | 0.62 | 1.23 | 0.97, 1.55 | 0.080 | 0.94 | 0.70, 1.28 | 0.60 | 1.26 | 0.94, 1.69 | 0.036 |
| Other | 1.32 | 0.74, 2.36 | | 1.82 | 0.91, 3.63 | | 1.45 | 0.62, 3.42 | | 3.37 | 1.18, 9.63 | |
| Long-Term Care Facility | 1.03 | 0.84, 1.27 | 0.75 | 0.92 | 0.71, 1.20 | 0.56 | 0.82 | 0.58, 1.15 | 0.25 | 1.89 | 1.40, 2.54 | <0.001 |
| Comorbidities | | | | | | | | | | | | |
| Diabetes | 1.04 | 0.87, 1.24 | 0.68 | 0.99 | 0.79, 1.25 | 0.96 | 0.94 | 0.70, 1.26 | 0.69 | 0.90 | 0.68, 1.20 | 0.47 |
| Hypertension | 0.87 | 0.69, 1.09 | 0.23 | 1.04 | 0.77, 1.40 | 0.81 | 0.86 | 0.59, 1.24 | 0.41 | 1.17 | 0.73, 1.89 | 0.51 |
| Chronic Kidney Disease | 0.88 | 0.72, 1.07 | 0.20 | 0.82 | 0.64, 1.06 | 0.13 | 1.06 | 0.77, 1.46 | 0.74 | 0.92 | 0.68, 1.25 | 0.59 |
| Cardiac Disease | 1.04 | 0.85, 1.26 | 0.71 | 1.20 | 0.93, 1.55 | 0.15 | 1.07 | 0.78, 1.47 | 0.69 | 1.14 | 0.83, 1.58 | 0.41 |
| Pulmonary Disease | 1.32 | 0.94, 1.86 | 0.11 | 1.41 | 0.90, 2.22 | 0.13 | 1.33 | 0.74, 2.41 | 0.34 | 1.27 | 0.70, 2.32 | 0.43 |
| Tobacco Use | 0.82 | 0.61, 1.11 | 0.20 | 0.64 | 0.43, 0.97 | 0.034 | 0.66 | 0.39, 1.10 | 0.11 | 0.67 | 0.39, 1.14 | 0.14 |
| Obesity | 0.91 | 0.73, 1.14 | 0.43 | 1.08 | 0.82, 1.44 | 0.58 | 0.83 | 0.57, 1.19 | 0.30 | 1.07 | 0.73, 1.58 | 0.72 |
| May 4 to July 25 Time Period | 1.05 | 0.89, 1.25 | 0.56 | 1.21 | 0.97, 1.51 | 0.093 | 0.93 | 0.70, 1.23 | 0.63 | 0.66 | 0.48, 0.91 | 0.011 |

Abbreviations: ICU=intensive-care unit; NIV=noninvasive ventilation; HR=hazard ratio; CI=confidence interval; REF=reference value

ORIGINAL UNEDITED MANUSCRIPT

## Figure Legends

**Figure 1. State Transitions Framework for Multi-state Analysis.** At each time point, patients were categorized into one of fifteen mutually exclusive and exhaustive states: (1) emergency department, (2) inpatient floor, (3) ICU admission without invasive mechanical ventilation, (4) noninvasive ventilation, (5) invasive mechanical ventilation in ICU, (6) noninvasive ventilation after invasive mechanical ventilation, (7) ICU after invasive mechanical ventilation, (8) inpatient floor after ICU admission but no invasive mechanical ventilation, (9) inpatient floor after invasive mechanical ventilation, (10) discharged without ICU admission, (11) discharged with history of ICU admission but no invasive mechanical ventilation, (12) discharged with history of invasive mechanical ventilation (IMV), (13) died, (14) died with history of ICU admission but no invasive mechanical ventilation, and (15) died with history of invasive mechanical ventilation. This figure depicts all the possible transitions patients could make from each state. Patients were not restricted to starting from State 1; those who were directly admitted to the hospital or transferred from another hospital started from the state in which they were first observed. Abbreviations: ICU=intensive-care unit; NIV=noninvasive ventilation; IMV=invasive mechanical ventilation.

**Figure 2. Longitudinal Outcomes among Hospitalized Patients with COVID-19.** These figures represent longitudinal outcomes among patients entering three specific care states from multi-state analyses. The figure depicts the proportion of patients estimated to be in each care state at any given time point accounting for the transitions patients made between different clinical states over time. Panel A depicts outcomes after patients are initially admitted to the hospital (n=1,577), Panel B depicts outcomes after patients are admitted to the ICU (n=571), Panel C depicts outcomes after noninvasive ventilation (n=343), and Panel D depicts outcomes after patients are intubated (n=214). Abbreviations: ED=Emergency Department; ICU=intensive-care unit; NIV=noninvasive ventilation; IMV=invasive mechanical ventilation.

**Figure 3. Trajectories of Hospitalized Patients with COVID-19 (n=1,417).** The alluvial plot depicts the trajectories of patients over their hospital course after admission. Alluviums are color-coded by patient outcome at 28 days and their width represents the number of patients. Only patients with 28 days of observation time were included (inclusive of time after discharge or death). Abbreviations: ICU=intensive-care unit; NIV=noninvasive ventilation; IMV=invasive mechanical ventilation.

**Figure 4. Hazard of ICU Admission, Noninvasive Ventilation, Intubation, Discharge, and Death Among Hospitalized Patients with COVID-19 (n=1,577).** This figure depicts the instantaneous hazard of ICU admission, noninvasive ventilation, intubation, discharge, and death at different timepoints since a patient was admitted initially admitted to the hospital. The table below represents numbers at risk from analyses stratified for each outcome. Abbreviations: ICU=intensive-care unit; NIV=noninvasive ventilation.

**Figure 5. Duration of Overall Hospitalization, ICU Admissions, Noninvasive Ventilation, and Invasive Mechanical Ventilation.** Violin plots represent the estimated overall duration of hospital admission from multi-state analyses from initial hospitalization to either discharge or death (Panel A), duration of ICU admission (Panel B), duration of noninvasive ventilation (Panel C), and duration invasive mechanical ventilation (Panel D). We present overall estimates as well as estimates stratified by populations with specific clinical outcomes. Dots represent the median values, the surrounding box spans between the $25^{th}$ and $75^{th}$ percentiles, and the surrounding violin plot represents a kernel density plot spanning the full range of values. Of note, kernel density plots extend below one due to estimation algorithms, but no patients had length of stays less than 0 in any state. Abbreviations: ICU=intensive-care unit; NIV=noninvasive ventilation; IMV=invasive mechanical ventilation.

**Figure 6. Transition Intensities from Inpatient Floor, ICU, Noninvasive Ventilation, and IMV Clinical States.** This figure depicts the instantaneous hazard of potential transitions from an initial starting clinical state to the next subsequent clinical state. X-axes represent the time since the patient initially entered a particular clinical state. Panel A depicts transitions after entering the Inpatient Floor state (i.e., state 2 to either state 3, 10, or 13), Panel B depicts transitions after entering the ICU state (i.e., state 3 to either state 4, 5, 8, or 14), Panel C depicts transitions after entering the Noninvasive Mechanical Ventilation state (i.e., state 4 to either state 5, 7, or 14), and Panel D depicts transitions after entering the Invasive Mechanical Ventilation state (i.e.,

ORIGINAL UNEDITED MANUSCRIPT

state 5 to either state 6, 7, or 15). Abbreviations: ICU=intensive-care unit; NIV=noninvasive ventilation; IMV=invasive mechanical ventilation.

**Figure 7. Forest Plots of Cumulative Incidence of ICU Admission, Noninvasive Ventilation, Intubation, and Death by 28 days Stratified by Patient Subgroups.** This figure depicts cumulative incidence of ICU admission (Panel A), noninvasive ventilation (Panel B), intubation (Panel C), or death (Panel D) by 28 days from stratified competing risk analyses using the Aalen-Johansen method. The reference line aligns with the estimate for the overall population.

**Figure 8. Adjusted Age-Stratified Estimates of ICU Admission, Noninvasive Ventilation, Intubation, and Death by Time Period.** This figure depicts adjusted age-stratified estimates of ICU admissions (Panel A), noninvasive ventilation (Panel B), intubation (Panel C), or death (Panel D) by secular time period. Marginal estimates were obtained from Poisson models adjusted for sex, race, patient comorbidities, and whether patients came from a long-term care facility with a time offset.

ORIGINAL UNEDITED MANUSCRIPT

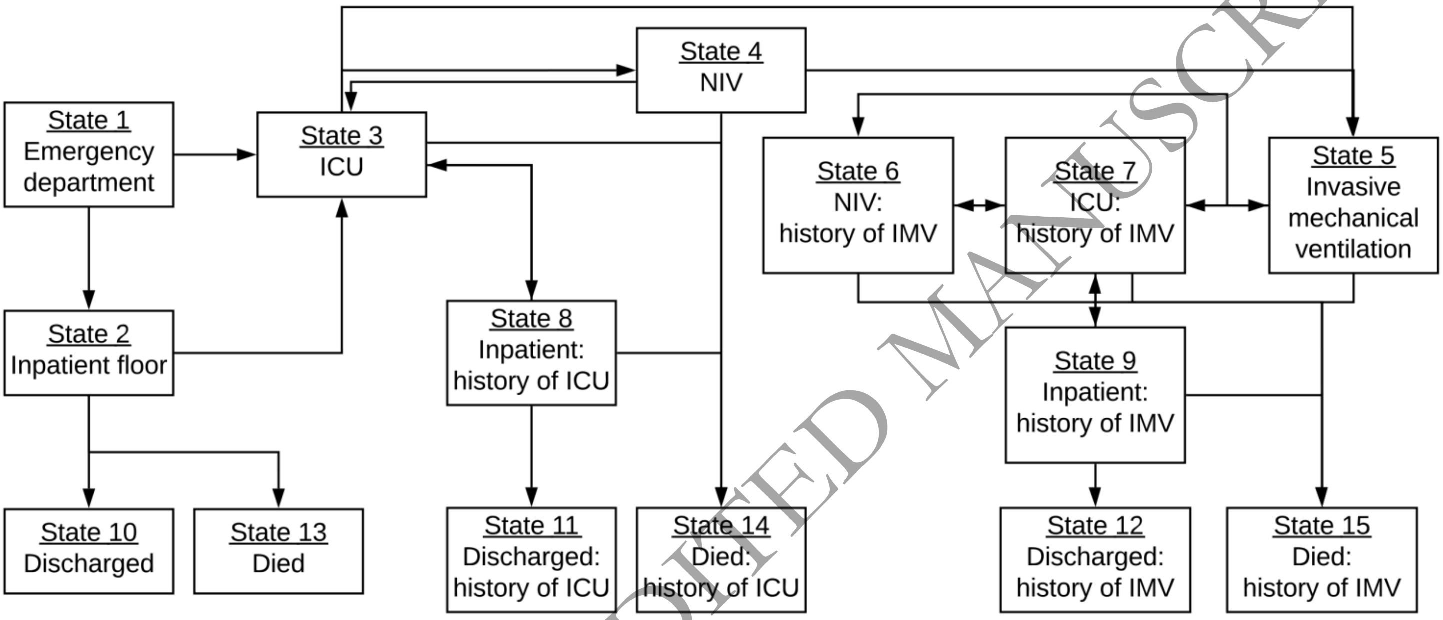

ORIGINAL UNEDITED MANUSCRIPT

ORIGINAL UNEDITED MANUSCRIPT

ORIGINAL UNEDITED MANUSCRIPT

ORIGINAL UNEDITED MANUSCRIPT

ORIGINAL UNEDITED MANUSCRIPT

ORIGINAL UNEDITED MANUSCRIPT

ORIGINAL UNEDITED MANUSCRIPT

ORIGINAL UNEDITED MANUSCRIPT

ORIGINAL UNEDITED MANUSCRIPT

ORIGINAL UNEDITED MANUSCRIPT

ORIGINAL UNEDITED MANUSCRIPT

ORIGINAL UNEDITED MANUSCRIPT

ORIGINAL UNEDITED MANUSCRIPT

ORIGINAL UNEDITED MANUSCRIPT

ORIGINAL UNEDITED MANUSCRIPT
