# The Clinical Course of Coronavirus Disease 2019 in a US Hospital System: A Multistate Analysis

**DOI:** 10.1093/aje/kwaa286

**Published:** 2020-12-22

**Authors:** Aaloke Mody, Patrick G Lyons, Cristina Vazquez Guillamet, Andrew Michelson, Sean Yu, Angella Sandra Namwase, Pratik Sinha, William G Powderly, Keith Woeltje, Elvin H Geng

**Keywords:** age-stratified mortality, clinical course, coronavirus disease 2019, COVID-19 hospitalizations, intensive care unit, longitudinal trajectory, mechanical ventilation, multistate analysis

## Abstract

There are limited data on longitudinal outcomes for coronavirus disease 2019 (COVID-19) hospitalizations that account for transitions between clinical states over time. Using electronic health record data from a hospital network in the St. Louis, Missouri, region, we performed multistate analyses to examine longitudinal transitions and outcomes among hospitalized adults with laboratory-confirmed COVID-19 with respect to 15 mutually exclusive clinical states. Between March 15 and July 25, 2020, a total of 1,577 patients in the network were hospitalized with COVID-19 (49.9% male; median age, 63 years (interquartile range, 50–75); 58.8% Black). Overall, 34.1% (95% confidence interval (CI): 26.4, 41.8) had an intensive care unit admission and 12.3% (95% CI: 8.5, 16.1) received invasive mechanical ventilation (IMV). The risk of decompensation peaked immediately after admission; discharges peaked around days 3–5, and deaths plateaued between days 7 and 16. At 28 days, 12.6% (95% CI: 9.6, 15.6) of patients had died (4.2% (95% CI: 3.2, 5.2) had received IMV) and 80.8% (95% CI: 75.4, 86.1) had been discharged. Among those receiving IMV, 35.1% (95% CI: 28.2, 42.0) remained intubated after 14 days; after 28 days, 37.6% (95% CI: 30.4, 44.7) had died and only 37.7% (95% CI: 30.6, 44.7) had been discharged. Multistate methods offer granular characterizations of the clinical course of COVID-19 and provide essential information for guiding both clinical decision-making and public health planning.

## Abbreviations

aHR: adjusted hazard ratio
CI: confidence interval
COVID-19: coronavirus disease 2019
ICU: intensive care unit
IMV: invasive mechanical ventilation
IQR: interquartile range
NIV: noninvasive ventilation
SARS-CoV-2: severe acute respiratory syndrome coronavirus 2

A careful characterization of the clinical course of coronavirus disease 2019 (COVID-19) during hospitalization will offer important insights into patients’ prognosis and the anticipated burden and duration of resources required for their care—basic clinical information which is still coming into focus for this novel pathogen. Hospitalized patients may take numerous pathways: Some only require brief stays, while others deteriorate and require admission to the intensive care unit (ICU), with or without invasive mechanical ventilation (IMV) (1–6). Even if these patients survive, many will experience protracted hospital courses prior to discharge. Deaths could occur immediately after admission or after decompensations later on in the hospitalization. An understanding of how patients transition through multiple clinical states over the course of their hospitalization—and the timing of these transitions—will offer situational awareness and information for clinical decision-making and public health planning as the epidemic continues to evolve.

To date, published data on the hospital course of COVID-19 do not yet provide a comprehensive descriptive picture indicative of the experience in the United States. For example, while case series do describe the number or incidence of deaths (1–6), such analyses have not captured information on movement between multiple clinical states over the course of hospitalization. Additionally, the rapidly evolving nature of the pandemic means that in many reports a substantial proportion of patients are still in the midst of their illness (7). These analyses have either presented cross-sectional estimates that do not account for this unequal follow-up time or have excluded patients with incomplete follow-up time, potentially creating bias in both scenarios (1–9). Furthermore, much of the early data on hospitalizations focused only on critically ill patients and came from single-center studies conducted earlier in the epidemic, largely from the worst-hit areas such as Wuhan, China (1–3), Lombardy, Italy (4), and New York, New York (5, 6), where outcomes may not be representative of outcomes elsewhere. Thus, more rigorous data from regions where the burden of COVID-19 did not exceed the capacity of health-care systems is needed to inform COVID-19 planning in the United States going forward.

To address these needs, we used data from the BJC HealthCare Hospital system in St. Louis, Missouri, and the surrounding regions to examine the totality of experience across a number of clinical conditions (e.g., inpatient floor admission, ICU stay, death, discharge) in a cohort of patients who were admitted with COVID-19. We used multistate methods to estimate the proportion of patients in various clinical conditions over time, as well as the amount of time spent in each state and rates of transition from each state. This analytical technique permits a more comprehensive examination of the cascade of outcomes (10) during COVID-19 hospitalizations for informing planning and policy.

**Figure 1:**
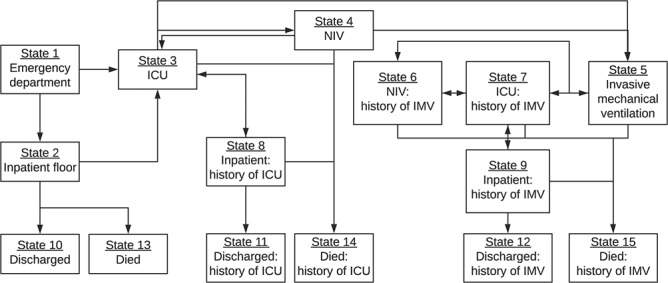
Framework for a multistate analysis of transitions between clinical states among hospitalized patients with coronavirus disease 2019. At each time point, patients were categorized into one of 15 mutually exclusive and exhaustive states: 1) emergency department, 2) inpatient floor, 3) intensive care unit (ICU) admission without invasive mechanical ventilation (IMV), 4) noninvasive ventilation (NIV), 5) IMV in the ICU, 6) NIV after IMV, 7) ICU after IMV, 8) inpatient floor after ICU admission but no IMV, 9) inpatient floor after IMV, 10) discharge without ICU admission, 11) discharge with a history of ICU admission but no IMV, 12) discharge with a history of IMV, 13) death, 14) death with a history of ICU admission but no IMV, and 15) death with a history of IMV. The figure depicts all of the possible transitions patients could make from each state. Patients were not restricted to starting from state 1; those who were directly admitted to the hospital or transferred from another hospital started from the state in which they were first observed.

**Table 1:** Baseline Characteristics of Coronavirus Disease 2019 Patients in the BJC HealthCare Hospital System (*n* = 1,577), St. Louis, Missouri, 2020

	**Clinical State**											
**Patient Characteristic**	**Inpatient (*n* = 1,577)**			**ICU (*n* = 571)**			**NIV (*n* = 343)**			**Intubation (*n* = 214)**		
	**No.** ^**a**^	**%**	**Median (IQR)**	**No.** ^**a**^	**%**	**Median (IQR)**	**No.** ^**a**^	**%**	**Median (IQR)**	**No.** ^**a**^	**%**	**Median (IQR)**
Male sex	787	49.9		330	57.8		198	57.7		131	61.2	
Age, years			63 (50–75)			65 (54–76)			66 (56–75)			65 (55–74)
Race/ethnicity												
Black or African-American	927	58.8		327	57.3		188	54.8		126	58.9	
White	571	36.2		210	36.8		138	40.2		73	34.1	
Other	30	1.9		21	3.7		8	2.3		9	4.2	
Unknown	49	3.1		13	2.3		9	2.6		6	2.8	
Long-term care facility resident	361	22.9		151	26.4		92	26.8		53	24.8	
Academic hospital	662	42.0		287	50.3		144	42.0		132	61.7	
Comorbid conditions^b^												
Diabetes mellitus	677	42.9		259	45.4		159	46.4		95	44.4	
Hypertension	1,190	75.5		436	76.4		272	79.3		164	76.6	
Chronic kidney disease	488	30.9		185	32.4		111	32.4		75	35.0	
Cardiac disease	687	43.6		265	46.4		170	49.6		97	45.3	
Pulmonary disease	481	30.5		184	32.2		113	32.9		60	28.0	
Tobacco use^b^	626	39.7		229	40.1		133	38.8		74	34.6	
Obesity^b^	849	53.8		300	52.5		188	54.8		103	48.1	
Baseline laboratory values^c^												
Hemoglobin level, g/dL	1,547		12.5 (11.0–13.8)	554		12.0 (10.2–13.4)	324		11.6 (9.7–13.2)	212		11.1 (9.6–13.0)
Platelet count, 10^3^ cells/mm^3^	1,547		208 (162–275)	554		202 (153–265)	324		210 (160–274)	212		210 (153–270)
White blood cell count, 10^3^ cells/mm^3^	1,547		7.0 (5.2–9.8)	554		8.0 (5.7–11.2)	324		8.0 (5.8–10.9)	212		10.1 (6.8–14.6)
Neutrophil count, 10^3^ cells/mm^3^	1,519		5.0 (3.4–7.6)	529		6.0 (4.0–9.2)	300		6.2 (4.1–9.2)	193		7.8 (5.4–11.2)
Lymphocyte count, 10^3^ cells/mm^3^	1,519		1.0 (0.8–1.6)	529		0.9 (0.7–1.3)	300		1.0 (0.7–1.3)	193		0.9 (0.6–1.4)
Creatinine level, mg/dL	1,527		1.1 (0.8–1.6)	547		1.2 (0.8–1.9)	320		1.1 (0.8–1.7)	205		1.3 (0.9–2.1)
Aspartate aminotransferase, units/L	1,365		44 (30–67)	498		54 (35–81)	282		56 (39–85)	187		65 (45–113)
Alanine aminotransferase, units/L	1,358		28 (18–45)	496		30 (20–50)	280		32 (21–56)	188		35 (23–65)
C-reactive protein, mg/L	716		84 (31–162)	332		124 (64–203)	198		137 (81–207)	123		160 (85–249)
Ferritin, ng/mL	636		597 (280–1,253)	300		762 (388–1,707)	178		800 (454–1,593)	119		1,131 (424–2,096)
D-dimer, ng/mL	698		1,075 (653–2,070)	327		1,370 (744–3,403)	195		1,200 (692–2,510)	117		1,861 (995–5,078)
Treatments												
Remdesivir	207	13.1		112	19.6		90	26.2		44	20.6	
Steroids^d^	408	25.9		238	41.7		172	50.1		115	53.7	
Tocilizumab	32	2.0		29	5.1		22	6.4		28	13.1	
Hydroxychloroquine	281	17.8		150	26.3		103	30.0		73	34.1	
Time period of hospitalization												
March 15–May 3	786	49.8		302	52.9		182	53.1		122	57.0	
May 4–July 25	791	50.2		269	47.1		161	46.9		92	43.0	

Abbreviations: ICU, intensive care unit; IQR, interquartile range; NIV, noninvasive ventilation.

^a^ Numbers may not sum to totals because of missing values.

^b^ Based on diagnosis codes from the electronic health record.

^c^ Baseline laboratory values were only included if laboratory tests were performed within 48 hours of either inpatient admission, ICU admission, or intubation, respectively.

^d^ Steroid equivalent to dexamethasone 6 mg/day.

## METHODS

### Study population and setting

We analyzed a consecutively compiled cohort of adult patients with confirmed COVID-19 who were admitted to the BJC HealthCare Hospital system between March 15, 2020, and July 25, 2020. We included all patients aged 18 years or older who were admitted to an inpatient service and either had a positive polymerase chain reaction test for severe acute respiratory syndrome coronavirus 2 (SARS-CoV-2) during admission or within the past 7 days or had confirmed COVID-19 disease as an encounter diagnosis. BJC HealthCare is a nonprofit health system that consists of 15 hospitals—ranging from a 1,200-bed academic referral center to a 40-bed rural community hospital—in the St. Louis, southern Illinois, and mid-Missouri regions. It serves a diverse population across the socioeconomic and sociodemographic spectra in both urban and rural regions, with a catchment area of approximately 3 million people (11). During the COVID-19 epidemic, BJC hospitals opened up additional ICUs to manage patients with COVID-19 but never exceeded health-care capacity with regard to hospital beds, ICU beds, mechanical ventilators, or staffing. General hospital management protocols are detailed in the Web Appendix (available at https://doi.org/10.1093/aje/kwaa286).

### Measurements

We extracted data for this analysis from electronic health records for the entire BJC system (Epic Systems Corporation, Verona, Wisconsin). Data collected included admission and discharge dates, sociodemographic information, laboratory results, diagnosis codes, level and mode of oxygen delivery, level of care (i.e., inpatient floor, ICU), procedures (i.e., intubation), and outcomes (i.e., death, discharge) for all patients as charted throughout their hospitalization in the electronic health record. As part of BJC’s routine and ongoing COVID-19 tracking efforts, all patients admitted with COVID-19 had their chart manually reviewed to determine whether they were a resident of a long-term care facility. Additionally, we performed targeted chart reviews (*n* = 27) to reconcile potential inconsistencies in COVID-19 diagnoses, level of care, and outcomes from the electronic health record data.

**Figure 2:**
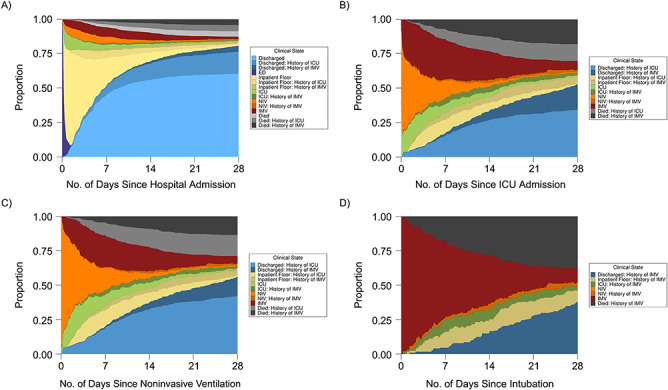
Longitudinal outcomes among hospitalized patients with coronavirus disease 2019 entering 3 specific clinical care states (multistate analyses), BJC HealthCare Hospital system, St. Louis, Missouri, 2020. The figure shows the proportion of patients estimated to be in each care state at any given time point, accounting for the transitions patients made between different clinical states over time. A) Outcomes following initial admission to a hospital (*n* = 1,577); B) outcomes following admission to the intensive care unit (ICU) (*n* = 571); C) outcomes following noninvasive ventilation (NIV) (*n* = 343); D) outcomes following intubation (*n* = 214). ED, emergency department; IMV, invasive mechanical ventilation.

**Figure 3:**
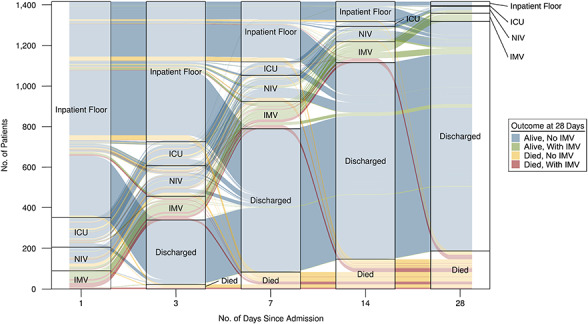
Clinical trajectories of hospitalized patients with coronavirus disease 2019 over the course of their hospital stay (*n* = 1,417), BJC HealthCare Hospital system, St. Louis, Missouri, 2020. Alluvia are color-coded by patient outcome at 28 days, and their width represents the number of patients. Only patients with 28 days of observation time were included (inclusive of time after discharge or death). ICU, intensive care unit; IMV, invasive mechanical ventilation; NIV, noninvasive ventilation.

**Figure 4:**
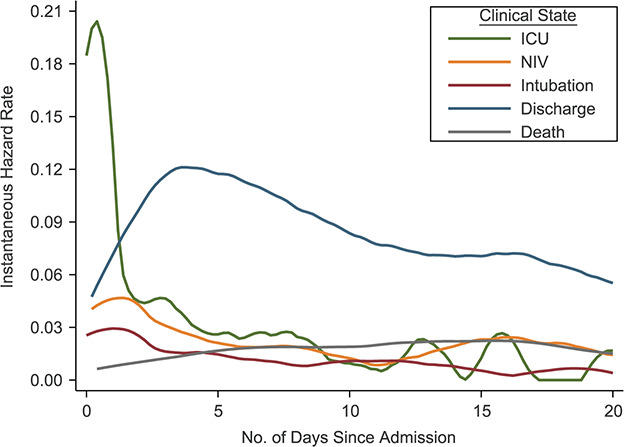
Instantaneous hazards of intensive care unit (ICU) admission, noninvasive ventilation (NIV), intubation, discharge, and death at different time points since admission among hospitalized patients with coronavirus disease 2019 (*n* = 1,577), BJC HealthCare Hospital system, St. Louis, Missouri, 2020.

**Figure 5:**
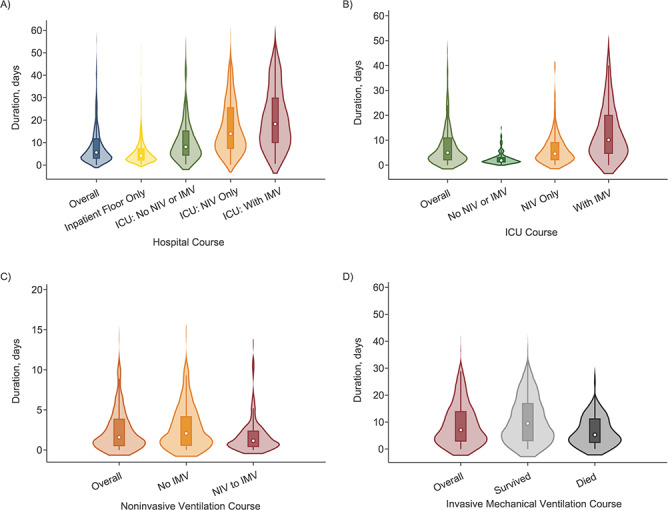
Estimated durations of overall patient stay (*n* = 1,577) (A), intensive care unit (ICU) stay (*n* = 571) (B), noninvasive ventilation (NIV) (*n* = 343) (C), and invasive mechanical ventilation (IMV) (*n* = 214) (D) among hospitalized patients with coronavirus disease 2019 (multistate analyses), BJC HealthCare Hospital system, St. Louis, Missouri, 2020. Dots represent the median values; the surrounding boxes span the 25th and 75th percentiles; and the violin plots show kernel density plots spanning the full range of values. Notably, kernel density plots extend below 1 because of estimation algorithms, but no patients had a length of stay less than 0 in any state.

**Figure 6:**
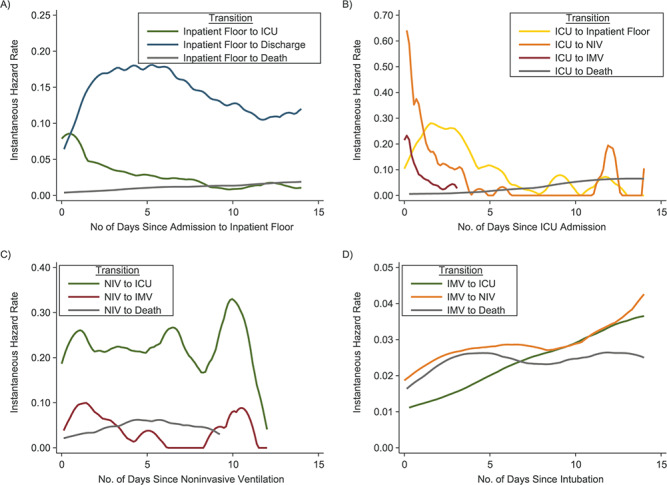
Transition intensities for transitions from the inpatient floor, intensive care unit (ICU), noninvasive ventilation (NIV), and invasive mechanical ventilation (IMV) clinical states among hospitalized patients with coronavirus disease 2019, BJC HealthCare Hospital system, St. Louis, Missouri, 2020. The figure depicts the instantaneous hazard of potential transitions from an initial starting clinical state to the next subsequent clinical state. Values shown on the *x*-axes represent the amount of time since the patient initially entered a particular clinical state. A) Transitions after entering the inpatient floor state (i.e., from state 2 to either state 3, 10, or 13) (*n* = 1,577); B) transitions after entering the ICU state (i.e., from state 3 to either state 4, 5, 8, or 14) (*n* = 571); C) transitions after entering the NIV state (i.e., from state 4 to either state 5, 7, or 14) (*n* = 343); D) transitions after entering the IMV state (i.e., from state 5 to either state 6, 7, or 15) (*n* = 214).

### Analyses

We sought to assess the clinical course of COVID-19 patients presenting to the hospital in a manner that accounted for the numerous changes in clinical status patients may have had over the duration of their hospitalization (e.g., admission, critical illness, intubation, death, discharge) (12–14). We first categorized patients into one of 15 mutually exclusive and exhaustive states based on their clinical status at each time point: 1) emergency department, 2) inpatient floor, 3) ICU admission without IMV, 4) noninvasive ventilation (NIV), 5) IMV in the ICU, 6) NIV after IMV, 7) ICU admission after IMV, 8) inpatient floor after ICU admission without IMV, 9) inpatient floor after IMV, 10) discharge without ICU admission, 11) discharge with a history of ICU admission without IMV, 12) discharge with a history of IMV, 13) death, 14) death with a history of ICU admission without IMV, and 15) death with a history of IMV (Figure 1). We then examined outcomes longitudinally in several ways to highlight unique aspects of patients’ clinical courses.

**Figure 7:**
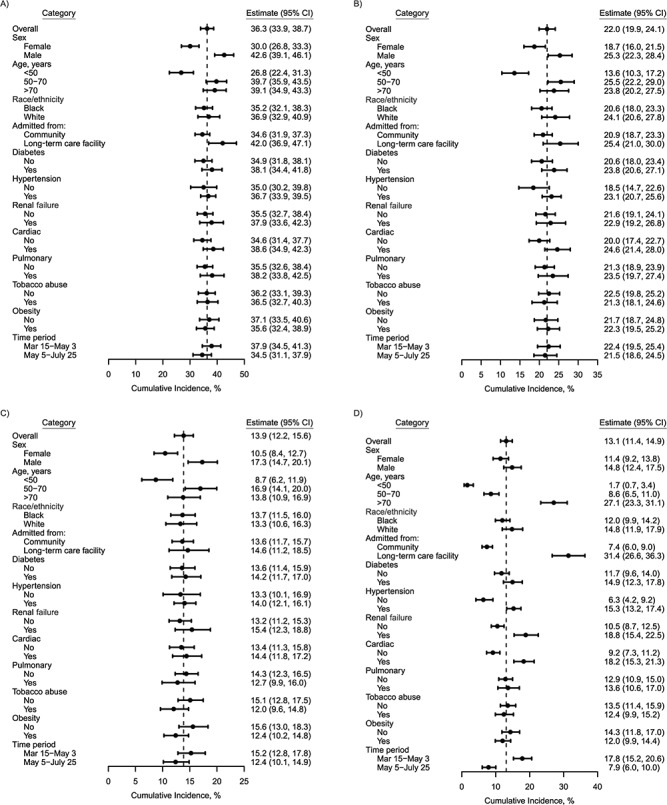
Cumulative incidence of intensive care unit admission (A), noninvasive ventilation (B), intubation (C), and death (D) by 28 days among hospitalized patients with coronavirus disease 2019, according to patient subgroup (*n* = 1,577), BJC HealthCare Hospital system, St. Louis, Missouri, 2020. Results were obtained in stratified competing-risk analyses using the Aalen-Johansen method. The reference line (vertical dashed line) aligns with the estimate for the overall population. Bars, 95% confidence intervals (CIs).

First, we applied nonparametric multistate analytical techniques based on the Aalen-Johansen method to account for patient movements into and out of multiple clinical states over time and for situations where the observation times for each patient were unequal (12–15). We estimated the probability over time of a patient’s having a particular clinical status after entering into one of 3 different states: 1) after inpatient admission, 2) after ICU admission, 3) after NIV, and 4) after endotracheal intubation. For each analysis, time 0 was the point of entry into that particular clinical state, and patients were censored at the time of discharge, death, or the end of the observation period (i.e., July 25, 2020).

Second, we estimated the instantaneous rates of ICU admission, NIV, intubation, death, and discharge after inpatient admission (regardless of movements through intermediate states) in order to characterize the dynamics of transitions between clinical states. Additionally, we also estimated transition intensities (i.e., instantaneous rate of transition to the next immediate state) after entering the inpatient floor (state 2), ICU (state 3), NIV (state 4), or IMV (state 5) state (Figure 1).

Third, we used an alluvial diagram to depict the trajectories of individual patients through clinical states over their hospitalization, stratifying by patients’ outcomes at 28 days. This analysis was restricted to patients with at least 28 days of observation (including time after death or discharge).

Fourth, we estimated the durations of overall hospitalization, ICU stays, NIV, and IMV based on results from the multistate analyses.

Fifth, we assessed the cumulative incidence of ICU admission, NIV, intubation, and death by 28 days since inpatient admission, stratifying by patient subgroups. We also performed Cox proportional hazards analyses to identify patient characteristics that were independently associated with times from inpatient admission to ICU admission, intubation, and death. We selected covariates using directed acyclic graphs based on a-priori hypotheses of causal relationships between baseline sociodemographic and clinical characteristics and patient outcomes. We evaluated the proportional hazards assumption using Schoenfeld residuals (16).

Lastly, to assess the changes in patient outcomes over time and explore the potential impact of the introduction of evidence-based therapies (i.e., remdesivir (17) and dexamethasone (18) in moderate or severe disease), we obtained adjusted age-stratified estimates of patient outcomes based on the time period in which they were admitted (i.e., March 15–May 3 (prior to remdesivir availability) or May 4–July 25 (after remdesivir availability)). We report these as marginal estimates from age-stratified Poisson models adjusting for sex, race/ethnicity, comorbidity, and whether the patient lived in a long-term care facility.

All analyses were conducted with R 3.2.4 software (R Foundation for Statistical Computing, Vienna, Austria) using the *mstate* package (13, 14) and Stata MP 16.1 (StataCorp LLC, College Station, Texas).

## RESULTS

### Patient characteristics

Between March 15 and July 25, 2020, a total of 2,940 patients who presented to an emergency department in the study area were confirmed to have COVID-19, and 1,577 were admitted to the hospital (Web Figure 1). Among those hospitalized, 571 patients were subsequently admitted to the ICU, 343 received NIV, and 214 received IMV (Table 1). The median age was 63 years (interquartile range (IQR), 50–75), and 927 patients (58.8%) were Black (Table 1). As the pandemic progressed, patients admitted later on were younger, had fewer comorbid conditions, were less likely to be Black, and were less likely to reside in a long-term care facility. They were more likely to be treated with remdesivir and steroids and less likely to be treated with tocilizumab and hydroxychloroquine (Web Table 1). Overall, Black patients tended to be younger and to have more comorbidity and were less likely to be male (Web Table 2).

### Clinical course of COVID-19 hospitalizations based on multistate analyses

Overall, 34.1% (95% confidence interval (CI): 26.4, 41.8) of hospitalized patients were in the ICU at some point during admission (including patients receiving IMV), and 12.3% (95% CI: 8.5, 16.1) received IMV (Figures 2 and 3, Web Table 3). After admission, the rates of transfer to the ICU and intubation peaked on hospital day 1 and declined thereafter, whereas the rate of discharge peaked between hospital days 3 and 5, and the rate of death plateaued on days 7 through 16 (Figure 4). At 7 days, 51.6% (95% CI: 47.5, 55.6) of patients had been discharged and 5.7% (95% CI: 3.7, 7.7) had died. At 28 days, 80.8% (95% CI: 75.4, 86.1) of patients (20.2% (95% CI: 17.4, 23.0) with a history of ICU admission and 4.3% (95% CI: 3.3, 5.3) with a history of IMV) had been discharged and 12.6% (95% CI: 9.6, 15.6) of patients (8.6% (95% CI: 6.6, 10.6) with an ICU admission and 4.2% (95% CI: 3.2, 5.2) with IMV) had died (Figure 2, Web Table 3). The median duration of hospital stay for all inpatient admissions was 5.7 days (IQR, 2.9–11.9). The median duration of hospital stay was 4.2 days (IQR, 2.1–7.4) for those cared for only on the inpatient floor, 8.1 days (IQR, 4.3–15.4) for those admitted to the ICU without receiving NIV or IMV, 14.1 days (IQR, 7.3–25.8) for who received NIV but no IMV, and 19.1 days (IQR, 10.1–30.7) for those who received IMV (Figure 5, Web Table 4).

Among patients admitted to the ICU and those who received NIV, 50.8% (95% CI: 35, 66.6) and 39.5% (95% CI: 26.6, 52.4) received IMV at some point, respectively (Figure 2, Web Table 3). The rates of noninvasive and invasive ventilation peaked immediately after ICU transfer, whereas the rate of death (without intubation) peaked around day 5, and the rate of transfer to the inpatient floor peaked on day 3 and again on day 12 (Figure 6). At 7 days after ICU admission, 53.9% (95% CI: 40, 67.8) of patients remained in the ICU (13.6% (95% CI: 9.4, 17.7) receiving NIV and 29.3% (95% CI: 23.8, 34.8) receiving IMV), 17.4% (95% CI: 11.5, 23.2) had been discharged from the hospital, and 14.3% (95% CI: 8.7, 19.9) had died (6.8% (95% CI: 4, 9.7) after IMV). At 28 days, 11.2% (95% CI: 5.2, 17.2) of patients remained in the ICU (6.5% (95% CI: 4.1, 9.0) receiving IMV), 52.9% (95% CI: 42.4, 63.4) had been discharged (18.4% (95% CI: 14.0, 22.8) had received IMV), and 30.3% (95% CI: 22.5, 38.0) had died (18.0% (95% CI: 13.5, 22.4) after IMV) (Figure 2, Web Table 3). The median duration of ICU admissions was 1.9 days (IQR, 1.1–3.2) without NIV or IMV, 4.5 days (IQR, 2.0–9.2) with NIV only, and 10.3 days (IQR, 4.6–20.1) for those who received IMV (Figure 5, Web Table 4).

Lastly, among patients who received IMV, the rate of extubation increased through day 14, while the hazard for death plateaued between days 5 and 12 (Figure 6). At 14 days after intubation, 35.1% (95% CI: 28.2, 42.0) of patients remained on IMV and 28.0% (95% CI: 21.1, 35.0) had died. At 28 days, 16.2% (95% CI: 8.2, 24.3) remained in the ICU (10.8% (95% CI: 6.7, 14.8) still receiving IMV), 37.6% (95% CI: 30.4, 44.7) had died, and only 37.7% (95% CI: 30.6, 44.7) had been discharged (Figure 2, Web Table 3). The median duration of IMV was 7.2 days (IQR, 2.9–14.2) (Figure 5, Web Table 4).

**Figure 8:**
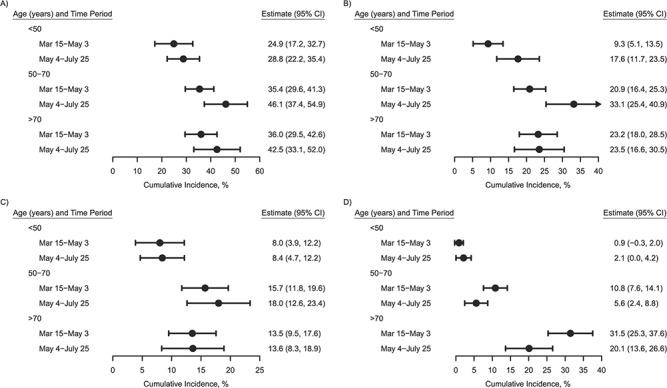
Age-stratified adjusted estimates of the cumulative incidence of intensive care unit admission (A), noninvasive ventilation (B), intubation (C), and death (D) among hospitalized patients with coronavirus disease 2019, by time period (*n* = 1,577), BJC HealthCare Hospital system, St. Louis, Missouri, 2020. Marginal estimates were obtained from Poisson models adjusting for sex, race/ethnicity, comorbidity, and whether the patient had come from a long-term care facility, with a time offset. Bars, 95% confidence intervals (CIs).

In stratified multistate and multivariable Cox proportional hazards analyses, older patients had markedly increased mortality (for age >70 years vs. <50 years, adjusted hazard ratio (aHR) = 7.00, 95% CI: 2.97, 16.48) and trended toward increased ICU admissions and receipt of NIV and IMV. Residents of long-term care facilities also had increased mortality (aHR = 1.89, 95% CI: 1.40, 2.54). Men were more likely than women to be admitted to the ICU (aHR = 1.53, 95% CI: 1.29, 1.81), to receive NIV (aHR = 1.34, 95% CI: 1.08, 1.66), and to receive IMV (aHR = 1.54, 95% CI: 1.16, 2.02) and potentially trended toward increased mortality. Patients with comorbidity trended toward increased mortality in stratified analyses but not multivariable analyses. Race/ethnicity was not significantly associated with ICU admission, NIV, IMV, or death. Lastly, being admitted between May 4 and July 25 (as opposed to earlier in the pandemic) was not associated with changes in the rate of ICU admission, NIV, or IMV but was associated with decreased mortality (aHR = 0.66, 95% CI: 0.48, 0.91) (Figure 7, Table 2, Web Tables 5 and 6). Decreases in mortality appeared greatest in older patients (Figure 8, Web Table 7).

## DISCUSSION

We used multistate analytical methods to longitudinally characterize the clinical course of COVID-19 disease after presentation to a hospital in a manner that accounted for patient transitions between multiple clinical states over the course of admission and the timing of these transitions. We found that at 7 days after hospital admission, 51.6% of patients had been discharged and 5.7% had died; at 28 days, 80.8% had been discharged (20.2% had been admitted to the ICU and 4.3% had received IMV) and 12.6% had died (8.6% had had an ICU admission and 4.2% had received IMV). The risk of decompensation was greatest immediately after admission; discharges peaked around days 3–5, and mortality plateaued between days 7 and 16. Among patients receiving IMV, 35.1% remained intubated and 28.0% had died after 14 days. Overall, these findings provide a more nuanced and comprehensive depiction of the trajectories of COVID-19 disease after presentation to the hospital.

Our study provides granular epidemiologic data on the clinical course of COVID-19 that are both essential for guiding public health officials in assessing their health systems’ capacity and immediately relevant for clinical decision-making (19). Early in the epidemic, one the primary concerns was the anticipated strain that unmitigated spread of SARS-CoV-2 was expected to place on health systems, and several influential disease models were built to specifically assess health systems’ capacity in terms of hospital beds and mechanical ventilators (20, 21). Our analysis details what happens to patients after being hospitalized with COVID-19—including during different phases of the pandemic—and can guide health systems in appropriately planning for the health-care resources that may be required. In particular, detailed data on the time spent in various clinical states can help researchers parameterize disease models to better project needs for staffing, hospital beds, critical-care beds, and mechanical ventilators (22–24). Additionally, it offers health-care providers a complete depiction of the trajectory the disease is likely to take based on a patient’s current clinical state and the probability of being in other clinical states at different time points further into hospitalization (e.g., in our study, patients received IMV for a median of only 7 days, but 28 days after intubation only 37.7% had been discharged, 37.6% had died, and 24.7% remained hospitalized). This level of granularity provides both public health officials and clinicians with valuable insights for guiding public health responses and making the most informed care decisions with patients and their families.

To our knowledge, our study is the first to have longitudinally characterized COVID-19 hospitalization trajectories in a way that comprehensively captures patient transitions between clinical-care states over time. Patients frequently transition between the inpatient floor, the ICU, and IMV—often more than once during a hospitalization—prior to discharge or death. To date, several studies have described COVID-19 hospitalizations (1, 3–6, 25), but most have focused only on critically ill patients and have provided cross-sectional estimates that included only patients with known outcomes and excluded patients who may have had prolonged hospitalizations and were still hospitalized (7). In one study that did include censored observations, the authors only considered time to a single outcome (i.e., in-hospital death); they did not consider intermediate events such as ICU transfers or intubation (5). Additionally, these estimates did not account for competing events (15), such as hospital discharge, that would preclude the occurrence of an in-hospital mortality event, potentially also contributing to bias (8, 9). Our study adds to this existing literature in several ways. We used rigorous longitudinal methods to estimate the incidence and timing of events in a setting where both competing events were present and where the observation times between participants were not equal (8, 9). Additionally, we used these multistate methods to assess transitions between multiple clinical states—as opposed to a single one—over the course of a patient’s hospitalization (13, 14). Furthermore, most early reports were single-center studies conducted in regions that had been hit hardest by COVID-19, potentially limiting the generalizability of patients’ experiences. In contrast, our data included a diverse and representative population from a variety of settings (e.g., both academic and community hospitals, rural and urban settings, affluent and marginalized communities) and information collected during different phases of the pandemic (i.e., before and after the introduction of evidence-based therapies). Thus, our study provides one of the most comprehensive characterizations of the clinical course of COVID-19 hospitalizations to date.

**Table 2:** Factors Associated With Intensive Care Unit Admission, Noninvasive Ventilation, Intubation, and Death Among Hospitalized Patients With Coronavirus Disease 2019 in Cox Proportional Hazards Regression Analysis (*n* = 1,577), BJC HealthCare Hospital System, St. Louis, Missouri, 2020

	**Clinical State**											
**Patient Characteristic**	**ICU Admission**			**NIV**			**Intubation**			**Death**		
	**Adjusted HR**	**95% CI**	***P* Value**	**Adjusted HR**	**95% CI**	***P* value**	**Adjusted HR**	**95% CI**	***P* value**	**Adjusted HR**	**95% CI**	***P* Value**
Male sex	1.53	1.29, 1.81	<0.001	1.34	1.08, 1.66	0.007	1.54	1.16, 2.02	0.002	1.20	0.91, 1.59	0.20
Age, years												
<50	1.00	Referent	0.006	1.00	Referent	0.010	1.00	Referent	0.012	1.00	Referent	<0.001
50–70	1.48	1.16, 1.89		1.62	1.17, 2.24		1.81	1.20, 2.74		2.91	1.23, 6.85	
>70	1.31	0.99, 1.73		1.33	0.91, 1.92		1.40	0.87, 2.25		7.00	2.97, 16.48	
Race/ethnicity												
Black	1.00	Referent	0.62	1.00	Referent	0.080	1.00	Referent	0.60	1.00	Referent	0.036
White	1.01	0.84, 1.21		1.23	0.97, 1.55		0.94	0.70, 1.28		1.26	0.94, 1.69	
Other	1.32	0.74, 2.36		1.82	0.91, 3.63		1.45	0.62, 3.42		3.37	1.18, 9.63	
Long-term care facility resident	1.03	0.84, 1.27	0.75	0.92	0.71, 1.20	0.56	0.82	0.58, 1.15	0.25	1.89	1.40, 2.54	<0.001
Comorbid conditions^a^												
Diabetes mellitus	1.04	0.87, 1.24	0.68	0.99	0.79, 1.25	0.96	0.94	0.70, 1.26	0.69	0.90	0.68, 1.20	0.47
Hypertension	0.87	0.69, 1.09	0.23	1.04	0.77, 1.40	0.81	0.86	0.59, 1.24	0.41	1.17	0.73, 1.89	0.51
Chronic kidney disease	0.88	0.72, 1.07	0.20	0.82	0.64, 1.06	0.13	1.06	0.77, 1.46	0.74	0.92	0.68, 1.25	0.59
Cardiac disease	1.04	0.85, 1.26	0.71	1.20	0.93, 1.55	0.15	1.07	0.78, 1.47	0.69	1.14	0.83, 1.58	0.41
Pulmonary disease	1.32	0.94, 1.86	0.11	1.41	0.90, 2.22	0.13	1.33	0.74, 2.41	0.34	1.27	0.70, 2.32	0.43
Tobacco use^a^	0.82	0.61, 1.11	0.20	0.64	0.43, 0.97	0.034	0.66	0.39, 1.10	0.11	0.67	0.39, 1.14	0.14
Obesity^a^	0.91	0.73, 1.14	0.43	1.08	0.82, 1.44	0.58	0.83	0.57, 1.19	0.30	1.07	0.73, 1.58	0.72
Hospitalization period May 4–July 25	1.05	0.89, 1.25	0.56	1.21	0.97, 1.51	0.093	0.93	0.70, 1.23	0.63	0.66	0.48, 0.91	0.011

Abbreviations: CI, confidence interval; HR, hazard ratio; ICU, intensive care unit; NIV, noninvasive ventilation.

^a^ Based on diagnosis codes from the electronic health record.

Our results offer an additional layer of nuance to characterizations of COVID-19-related hospitalizations but are also consistent with what has been previously reported (1–6, 26, 27). The majority of patients were admitted to the inpatient floor and discharged within 3–5 days, but an important subset of COVID-19 patients present critically ill (or decompensate early in their hospitalization) and generally experience a protracted hospital course, often with prolonged periods of IMV and a high risk for mortality. In our cohort, older age was most strongly associated with poor outcomes such as a need for IMV and mortality, followed by male sex. Additionally, we found that patients admitted after May 4, 2020 (i.e., after remdesivir was introduced in our hospital network) had reduced mortality rates, though patients admitted during this period were also substantially younger and healthier. Still, this association remained even after adjustment for age and comorbidity and may thus also be indicative—though not definitively so—of the positive impact of routine use of these evidence-based therapies (i.e., remdesivir (17) and dexamethasone (18)) for COVID-19, particularly in older patients. Third, Black patients comprised a greater proportion of those admitted with COVID-19 disease, but, once hospitalized, there were no significant differences in outcomes in adjusted models. This is in line with prior studies and can probably be explained by the systemic disparities that have led to higher risks of acquiring COVID-19 in Black communities (27–32) but limited differences in the actual pathophysiology of the disease once a person becomes infected. Fourth, there were trends toward increased mortality with additional comorbidity in stratified analyses, but this was not consistent in multivariable regression. Lastly, though outcome estimates are also similar to those for influenza-associated and general acute respiratory distress syndrome (33, 34), more work is needed to understand how COVID-19 clinical phenotypes relate to their underlying pathophysiology and how they differ from other disease states (35–38). Ultimately, further research extending these findings is needed to help us understand for whom, when, and what types of interventions and treatments are needed for optimizing our response to COVID-19, at both the individual patient and public health levels.

There were several limitations to this study. First, we leveraged observational electronic health record data, which may have misclassified some patient outcomes, COVID-19 diagnoses, hospital events, or their timing. In particular, we did not have granular data on patients’ disease severity (e.g., oxygenation levels), the exact timing of multiple events occurring within an hour of each other, or the history or circumstances leading up to admission at a BJC hospital (e.g., duration of symptoms, prior events if the patient transferred from a different hospital). Second, we obtained adjusted age-stratified outcome estimates by time period to explore the potential impact of routine use of evidence-based COVID-19 therapies, but these analyses were not adjusted for disease severity at initial patient presentation, and it is still possible that these estimates were affected by residual confounding. Third, our study included only hospitals from a large health system affiliated with an academic medical center where health-care capacity was not exceeded, and it may not necessarily be reflective of outcomes in other regions of the country or the world, particularly places that experienced a COVID-19 epidemic surge that exceeded their health systems’ capacity. Still, we did include patients from several hospitals ranging from an academic, quaternary-care medical center to smaller community hospitals located in both urban and rural settings.

In conclusion, we used multistate analytical methods to provide nuanced characterizations of the clinical course of COVID-19 hospitalizations. Multistate approaches provide granular descriptions of patients’ trajectories over time and offer useful insights on COVID-19 disease for front-line clinicians, disease modelers, and health system and public health officials.

## Supplementary Material

Web_Material_kwaa286
