# Supplementary material for: The Clinical Course of Coronavirus Disease 2019 in a US Hospital System: A Multistate Analysis
Source: Am J Epidemiol. 2020 Dec 22;190(4):539–52. doi: 10.1093/aje/kwaa286 (PMC7799307; doi:10.1093/aje/kwaa286)
Supplement: Web_Material_kwaa286 [file web_material_kwaa286.pdf]

WEB MATERIAL

The Clinical Course of Coronavirus Disease 2019 in a US Hospital System: A Multistate Analysis

Aaloke Mody, Patrick G. Lyons, Cristina Vazquez Guillamet, Andrew Michelson, Sean Yu, Angella Sandra Namwase, Pratik Sinha, William G. Powderly, Keith Woeltje, and Elvin H. Geng

Table of Contents

Web Appendix. Management of Patients with COVID-19 at BJC Hospitals……………….……………2

Web Table 1. Baseline Patient Characteristics Stratified by Time Period……………………..………4

Web Table 2. Baseline Patient Characteristics Stratified by Race………………………………..……6

Web Table 3. Multistate Estimates of the Proportion of Patients in Each Care State at a Given Time Point……………………..…………….....…………………………………………………………………8

Web Table 4. Multistate Estimates Stratified by Time Period……………………………………….…10

Web Table 5. Length of Stay Estimates based on Multistate Analyses…………………………………14
..

Web Table 6. Stratified Estimates of the Cumulative Incidence of ICU Admission, Intubation, and Death by 28 days………………………………………………………………………………………………15

Web Table 7. Adjusted Age-Stratified Estimates of ICU Admission, Noninvasive Ventilation, Intubation, and Death by Time Period…………………………………………………………….……16

Web Figure 1. Daily Number of New Inpatient Admissions, ICU admissions, and Intubations…………………………………………………………………………………………...……17

WEB APPENDIX

Management of Patients With COVID-19 at BJC Hospitals

Bed geography and patient cohorting

Across BJC hospitals during the study period, patients with diagnosed or suspected COVID-19 were hospitalized in private rooms, and were grouped in geographically distinct ward and intensive care units whenever possible. At Barnes-Jewish Hospital, BJC's quaternary referral center (which was responsible for 42% of the COVID-19 admissions system-wide), COVID-19 patients and patients under investigation received all their care in one of several dedicated COVID-19 wards or ICUs.

Management of respiratory failure

At the beginning of the pandemic, system-wide guidance on management of respiratory failure in patients with diagnosed or suspected COVID-19 included recommendations to:

1. avoid potentially-aerosolizing modalities of respiratory support (i.e., humidified high-flow nasal cannula [HHFNC] or non-invasive positive pressure ventilation [NIPPV]);

2. avoid the risk of acute respiratory deterioration by considering early rapid-sequence intubation [RSI] and invasive mechanical ventilation [IMV] for patients receiving > 6 liters per minute [LPM] of oxygen or with increased work of breathing;

3. use standard, evidence-based strategies for management of acute hypoxemic respiratory failure [AHRF], including lung-protective ventilation (1), conservative fluid balance (2), prone positioning for appropriate patients (3), and consideration of neuromuscular blockade (4), inhaled pulmonary vasodilators (5, 6), and extracorporeal membrane oxygenation for refractory hypoxemia (7); and

4. use standard, evidence-based strategies for weaning and liberation from IMV, including daily spontaneous breathing trials and sedation holidays.

On April 10, system-wide guidance was updated to reflect an evolving understanding of the safety and effectiveness of nonventilator strategies for appropriately-selected patients with known or suspected COVID-19-related respiratory failure. Important changes to recommended practice included:

1. Consideration of HHFNC, NIPPV, and inhaled pulmonary vasodilators for appropriately-selected patients with AHRF (i.e., reasonable work of breathing, ventilation, and mental status); and

2. Consideration of an “expectant management” approach, rather than intubation, for patients with AHRF requiring > 6 LPM oxygen.

System-wide recommendations for tracheostomy placement for patients with AHRF and known or suspected COVID-19 balanced published guidelines with the need to minimize healthcare worker exposure to the virus during a potentially-aerosolizing procedure. Consideration of tracheostomy was given to patients unable to be liberated from the ventilator after 14-21 days of IMV. Decisions regarding percutaneous versus surgical tracheostomy were left to the discretion of the clinical team.

Ventilator sharing was not performed at BJC during the study period.

Pharmacologic therapies

System-wide guidance for pharmacologic therapy with potential to act directly against SARS-CoV-2 (e.g., antiviral therapy) or specific complications of COVID-19, was as follows:

- *Remdesivir:* Available for use under Gilead's expanded access program on May 4, 2020 and then through the FDA's emergency use access program beginning May 14, 2020; prior to this it was unavailable including through clinical trials. available system-wide starting May 4, 2020; not available prior to this date

- *Hydroxychloroquine:* Recommended against use on April 28, 2020; prior to this recommendations were to its use in patients with COVID-19.

- *Corticosteroids:* Up to provider discretion

- *Tocilizumab:* Use of tocilizumab was initially left to provider discretion, but its use was explicitly recommended against on March 24, 2020.

- *Empiric anticoagulation:* Up to provider discretion, unless a clear indication for anticoagulation (e.g., known or suspected venous thromboembolism). Thrombolytic therapy was recommended against throughout the study period

References

1. Fan E, Del Sorbo L, Goligher EC, et al. An Official American Thoracic Society/European Society of Intensive Care Medicine/Society of Critical Care Medicine Clinical Practice Guideline: Mechanical Ventilation in Adult Patients with Acute Respiratory Distress Syndrome. *Am J Respir Crit Care Med* 2017;195(9):1253-63.
2. Mikkelsen ME, Christie JD, Lanken PN, et al. The adult respiratory distress syndrome cognitive outcomes study: long-term neuropsychological function in survivors of acute lung injury. *Am J Respir Crit Care Med* 2012;185(12):1307-15.
3. Guerin C, Reignier J, Richard JC, et al. Prone positioning in severe acute respiratory distress syndrome. *N Engl J Med* 2013;368(23):2159-68.
4. National Heart L, Blood Institute PCTN, Moss M, et al. Early Neuromuscular Blockade in the Acute Respiratory Distress Syndrome. *N Engl J Med* 2019;380(21):1997-2008.
5. Afshari A, Brok J, Moller AM, et al. Inhaled nitric oxide for acute respiratory distress syndrome (ARDS) and acute lung injury in children and adults. *Cochrane Database Syst Rev* 2010(7):CD002787.
6. Afshari A, Brok J, Moller AM, et al. Aerosolized prostacyclin for acute lung injury (ALI) and acute respiratory distress syndrome (ARDS). *Cochrane Database Syst Rev* 2010(8):CD007733.
7. Combes A, Hajage D, Capellier G, et al. Extracorporeal Membrane Oxygenation for Severe Acute Respiratory Distress Syndrome. *N Engl J Med* 2018;378(21):1965-75.

Web Table 1. Baseline Patient Characteristics Stratified by Time Period (*n* = 1,577)

	Inpatient		ICU		NIV		Intubated	
	**Mar 15-May 3 (n=786)**	**May 4-July 25 (n=791)**	**Mar 15-May 3 (n=302)**	**May 4-July 25 (n=269)**	**Mar 15-May 3 (n=182)**	**May 4-July 25 (n=161)**	**Mar 15-May 3 (n=122)**	**May 4-July 25 (n=92)**
Male Sex, n (%)	399 (50.8%)	388 (49.1%)	186 (61.6%)	144 (53.5%)	112 (61.5%)	86 (53.4%)	79 (64.8%)	52 (56.5%)
Median Age, years (IQR)	65 (55, 77)	60 (45, 73)	67 (57, 78)	62 (49, 73)	68 (58, 78)	63 (51, 73)	66 (57, 74)	62 (51, 72)
Race, n (%)								
Black or African American	502 (63.9%)	425 (53.7%)	188 (62.3%)	139 (51.7%)	103 (56.6%)	85 (52.8%)	78 (63.9%)	48 (52.2%)
White	262 (33.3%)	309 (39.1%)	104 (34.4%)	106 (39.4%)	75 (41.2%)	63 (39.1%)	39 (32.0%)	34 (37.0%)
Other	5 (0.6%)	25 (3.2%)	1 (0.3%)	12 (4.5%)	0 (0.0%)	9 (5.6%)	1 (0.8%)	5 (5.4%)
Unknown	17 (2.2%)	32 (4.0%)	9 (3.0%)	12 (4.5%)	4 (2.2%)	4 (2.5%)	4 (3.3%)	5 (5.4%)
Long-Term Care Facility, n (%)	224 (28.5%)	137 (17.3%)	96 (31.8%)	55 (20.4%)	61 (33.5%)	31 (19.3%)	33 (27.0%)	20 (21.7%)
Academic Hospital, n (%)	317 (40.3%)	345 (43.6%)	165 (54.6%)	122 (45.4%)	77 (42.3%)	67 (41.6%)	77 (63.1%)	55 (59.8%)
Diabetes, n (%)	345 (43.9%)	332 (42.0%)	137 (45.4%)	122 (45.4%)	84 (46.2%)	75 (46.6%)	53 (43.4%)	42 (45.7%)
Hypertension, n (%)	619 (78.8%)	571 (72.2%)	244 (80.8%)	192 (71.4%)	149 (81.9%)	123 (76.4%)	95 (77.9%)	69 (75.0%)
Chronic Kidney Disease, n (%)	263 (33.5%)	225 (28.4%)	109 (36.1%)	76 (28.3%)	63 (34.6%)	48 (29.8%)	43 (35.2%)	32 (34.8%)
Cardiac Disease, n (%)	365 (46.4%)	322 (40.7%)	146 (48.3%)	119 (44.2%)	95 (52.2%)	75 (46.6%)	56 (45.9%)	41 (44.6%)
Pulmonary Disease, n (%)	247 (31.4%)	234 (29.6%)	94 (31.1%)	90 (33.5%)	57 (31.3%)	56 (34.8%)	32 (26.2%)	28 (30.4%)
Tobacco use, n (%)	312 (39.7%)	314 (39.7%)	119 (39.4%)	110 (40.9%)	72 (39.6%)	61 (37.9%)	41 (33.6%)	33 (35.9%)
Obesity, n (%)	424 (53.9%)	425 (53.7%)	157 (52.0%)	143 (53.2%)	97 (53.3%)	91 (56.5%)	63 (51.6%)	40 (43.5%)
Median hemoglobin level, g/dl (IQR)	12.5 (11.0, 13.8)	12.5 (10.9, 13.9)	11.7 (10.2, 13.4)	12.1 (10.2, 13.5)	11.2 (9.7, 13.0)	11.9 (9.8, 13.2)	11.5 (9.8, 13.1)	10.7 (8.9, 12.5)
Median platelet count, $10^3/mm^3$ (IQR)	202 (159, 263)	215 (164, 287)	196 (148, 261)	212 (160, 268)	215 (161, 275)	204 (159, 272)	198 (152, 256)	238 (158, 313)
Median white blood cell count, $10^3/mm^3$ (IQR)	6.8 (5.0, 9.5)	7.1 (5.3, 10.3)	8.1 (5.6, 10.8)	7.9 (5.7, 11.9)	8.0 (5.5, 10.5)	8.0 (6.1, 11.9)	8.9 (6.4, 12.8)	11.1 (7.7, 15.1)
Median neutrophil count, $10^3/mm^3$ (IQR)	5.0 (3.4, 7.3)	5.0 (3.4, 7.8)	5.9 (3.9, 8.7)	6.1 (4.0, 10.1)	5.8 (3.8, 8.4)	6.2 (4.6, 9.8)	7.1 (5.3, 9.7)	9.5 (6.1, 12.0)
Median lymphocyte count, $10^3/mm^3$ (IQR)	1.0 (0.7, 1.4)	1.1 (0.8, 1.6)	0.9 (0.7, 1.3)	1.0 (0.7, 1.5)	1.0 (0.7, 1.3)	0.9 (0.6, 1.3)	0.9 (0.6, 1.3)	1.0 (0.7, 1.6)
Median creatinine level, mg/dL (IQR)	1.1 (0.8, 1.7)	1.0 (0.8, 1.5)	1.2 (0.9, 2.0)	1.1 (0.8, 1.7)	1.1 (0.8, 1.7)	1.0 (0.8, 1.6)	1.3 (1.0, 2.0)	1.3 (0.9, 2.4)
Median aspartate aminotransferase, units/L (IQR)	47 (32, 69)	41 (29, 63)	57 (39, 85)	49 (33, 77)	58 (42, 84)	50 (35, 90)	65 (48, 116)	62 (42, 108)

Median alanine aminotransferase, units/L (IQR)	28 (18, 45)	27 (18, 44)	32 (20, 53)	29 (20, 47)	31 (21, 56)	33 (22, 58)	39 (25, 64)	29 (21, 70)
Median C-reactive protein, mg/L (IQR)	95 (41, 162)	78 (24, 153)	128 (74, 201)	113 (47, 206)	131 (85, 189)	143 (81, 220)	152 (98, 222)	162 (69, 270)
Median ferritin, ng/mL (IQR)	626 (328, 1332)	538 (247, 1225)	800 (490, 1659)	733 (304, 1736)	1001 (600, 1621)	735 (374, 1501)	1092 (614, 2101)	1161 (325, 2096)
Median D-dimer, ng/mL (IQR)	1209 (723, 2416)	1007 (640, 1929)	1501 (875, 3403)	1122 (655, 2542)	1477 (860, 3140)	988 (651, 2058)	1944 (919, 4224)	1790 (1251, 7496)
Remdesivir, n (%)	1 (0.1%)	206 (26.0%)	1 (0.3%)	111 (41.3%)	1 (0.5%)	89 (55.3%)	1 (0.8%)	43 (46.7%)
Steroids*, n (%)	151 (19.2%)	257 (32.5%)	93 (30.8%)	145 (53.9%)	66 (36.3%)	106 (65.8%)	49 (40.2%)	66 (71.7%)
Tocilizumab, n (%)	25 (3.2%)	7 (0.9%)	23 (7.6%)	6 (2.2%)	16 (8.8%)	6 (3.7%)	22 (18.0%)	6 (6.5%)
Hydroxychloroquine, n (%)	268 (34.1%)	11 (1.4%)	148 (49.0%)	2 (0.7%)	101 (55.5%)	2 (1.2%)	73 (59.8%)	0 (0.0%)

Footnote: Baseline laboratory values were only included if they were 48 hours of either inpatient admission, ICU admission, or intubation, respectively.

*Steroid equivalent to dexamethasone 6 mg per day.

Abbreviations: ICU, intensive care unit; NIV, noninvasive ventilation.

Web Table 2. Baseline Patient Characteristics Stratified by Race (*n* = 1,500)

	Inpatient		ICU		NIV		Intubated	
	Black (n=927)	White (n=573)	Black (n=327)	White (n=210)	Black (n=188)	White (n=138)	Black (n=126)	White (n=73)
Male Sex, n (%)	435 (46.9%)	300 (52.4%)	175 (53.5%)	134 (63.8%)	100 (53.2%)	87 (63.0%)	72 (57.1%)	50 (68.5%)
Median Age, years (IQR)	61 (49, 73)	68 (56, 80)	63 (53, 74)	68 (59, 79)	65 (54, 74)	68 (60, 79)	65 (54, 73)	67 (57, 75)
Long-Term Care Facility, n (%)	199 (21.5%)	154 (26.9%)	85 (26.0%)	60 (28.6%)	53 (28.2%)	35 (25.4%)	34 (27.0%)	17 (23.3%)
Academic Hospital, n (%)	428 (46.2%)	175 (30.5%)	185 (56.6%)	77 (36.7%)	93 (49.5%)	39 (28.3%)	84 (66.7%)	36 (49.3%)
Diabetes, n (%)	436 (47.0%)	218 (38.0%)	171 (52.3%)	78 (37.1%)	103 (54.8%)	49 (35.5%)	69 (54.8%)	23 (31.5%)
Hypertension, n (%)	739 (79.7%)	414 (72.3%)	269 (82.3%)	152 (72.4%)	161 (85.6%)	103 (74.6%)	101 (80.2%)	52 (71.2%)
Chronic Kidney Disease, n (%)	320 (34.5%)	163 (28.4%)	114 (34.9%)	68 (32.4%)	70 (37.2%)	41 (29.7%)	50 (39.7%)	23 (31.5%)
Cardiac Disease, n (%)	403 (43.5%)	276 (48.2%)	144 (44.0%)	116 (55.2%)	91 (48.4%)	75 (54.3%)	58 (46.0%)	36 (49.3%)
Pulmonary Disease, n (%)	293 (31.6%)	180 (31.4%)	99 (30.3%)	80 (38.1%)	59 (31.4%)	51 (37.0%)	28 (22.2%)	31 (42.5%)
Tobacco use, n (%)	402 (43.4%)	214 (37.3%)	136 (41.6%)	88 (41.9%)	75 (39.9%)	55 (39.9%)	42 (33.3%)	31 (42.5%)
Obesity, n (%)	520 (56.1%)	314 (54.8%)	172 (52.6%)	122 (58.1%)	104 (55.3%)	80 (58.0%)	56 (44.4%)	46 (63.0%)
Median hemoglobin level, g/dl (IQR)	12.4 (10.9, 13.6)	12.6 (11.2, 14.0)	12.0 (10.3, 13.4)	11.9 (10.1, 13.4)	11.8 (9.5, 13.1)	11.4 (9.9, 13.1)	10.9 (9.2, 13.1)	11.4 (9.8, 12.6)
Median platelet count, $10^3/mm^3$ (IQR)	212 (165, 282)	199 (157, 261)	211 (156, 274)	191 (148, 254)	218 (161, 288)	191 (158, 252)	218 (170, 294)	176 (130, 252)
Median white blood cell count, $10^3/mm^3$ (IQR)	6.9 (5.2, 9.4)	7.0 (5.1, 10.1)	8.1 (5.8, 11.3)	7.4 (5.1, 10.8)	8.3 (6.1, 11.4)	7.6 (5.4, 10.4)	9.9 (7.3, 14.7)	9.0 (5.9, 14.2)
Median neutrophil count, $10^3/mm^3$ (IQR)	4.9 (3.3, 7.2)	5.1 (3.4, 8.1)	6.1 (4.1, 9.1)	5.4 (3.9, 8.8)	6.2 (4.4, 9.5)	5.8 (4.1, 8.1)	7.6 (5.6, 10.6)	7.1 (4.7, 11.6)
Median lymphocyte count, $10^3/mm^3$ (IQR)	1.1 (0.8, 1.7)	0.9 (0.6, 1.4)	1.0 (0.7, 1.5)	0.8 (0.6, 1.2)	1.1 (0.7, 1.5)	0.9 (0.6, 1.2)	1.0 (0.7, 1.6)	0.8 (0.6, 1.1)
Median creatinine level, mg/dL (IQR)	1.1 (0.8, 1.8)	1.0 (0.8, 1.4)	1.2 (0.9, 2.0)	1.1 (0.8, 1.6)	1.1 (0.9, 1.8)	1.0 (0.8, 1.5)	1.4 (1.0, 2.3)	1.3 (0.8, 1.9)
Median aspartate aminotransferase, units/L (IQR)	46 (32, 70)	40 (28, 58)	57 (38, 85)	46 (32, 70)	63 (44, 93)	44 (34, 68)	66 (48, 116)	62 (40, 97)
Median alanine aminotransferase, units/L (IQR)	28 (18, 44)	27 (18, 44)	29 (20, 47)	29 (18, 53)	33 (23, 50)	29 (19, 52)	35 (22, 58)	32 (23, 66)
Median C-reactive protein, mg/L (IQR)	85 (29, 167)	80 (32, 149)	129 (69, 208)	123 (56, 188)	136 (76, 198)	145 (92, 238)	185 (86, 263)	128 (95, 216)
Median ferritin, ng/mL (IQR)	616 (298, 1437)	550 (246, 960)	760 (428, 1904)	779 (297, 1588)	898 (492, 1570)	790 (498, 1468)	913 (486, 2162)	1351 (424, 2216)
Median D-dimer, ng/mL (IQR)	1168 (698, 2110)	1002 (635, 1830)	1454 (758, 3823)	1136 (737, 3080)	1233 (723, 2211)	1200 (695, 2683)	2140 (1259, 5566)	1402 (893, 3403)

Remdesivir, n (%)	106 (11.4%)	83 (14.5%)	57 (17.4%)	42 (20.0%)	48 (25.5%)	31 (22.5%)	22 (17.5%)	16 (21.9%)
Steroids*, n (%)	240 (25.9%)	142 (24.8%)	142 (43.4%)	77 (36.7%)	105 (55.9%)	56 (40.6%)	66 (52.4%)	37 (50.7%)
Tocilizumab, n (%)	15 (1.6%)	14 (2.4%)	13 (4.0%)	13 (6.2%)	7 (3.7%)	13 (9.4%)	13 (10.3%)	12 (16.4%)
Hydroxychloroquine, n (%)	178 (19.2%)	96 (16.8%)	92 (28.1%)	56 (26.7%)	55 (29.3%)	47 (34.1%)	48 (38.1%)	24 (32.9%)
Time Period, n (%)								
March 15 – May 3	502 (54.2%)	263 (45.9%)	188 (57.5%)	104 (49.5%)	103 (54.8%)	75 (54.3%)	78 (61.9%)	39 (53.4%)
May 4 – July 25	425 (45.8%)	310 (54.1%)	139 (42.5%)	106 (50.5%)	85 (45.2%)	63 (45.7%)	48 (38.1%)	34 (46.6%)

Footnote: Baseline laboratory values were only included if they were 48 hours of either inpatient admission, ICU admission, or intubation, respectively.

*Steroid equivalent to dexamethasone 6 mg per day.

Abbreviations: ICU, intensive care unit; NIV, noninvasive ventilation.

Web Table 3. Multistate Estimates of the Proportion of Patients in Each Care State at a Given Time Point (*n* = 1,577)

	**Inpatient Floor**	**ICU**	**NIV**	**IMV**	**NIV – Hx of IMV**	**ICU – Hx of IMV**	**Inpatient Floor – Hx of ICU**	**Inpatient Floor – Hx of IMV**
Days since Hospital Admission, % (95% CI)								
7	20.6 (18.5-22.6)	3.6 (2.7-4.6)	4.6 (3.6-5.7)	6.9 (5.7-8.2)	0.2 (0.0-0.4)	0.9 (0.4-1.3)	5.0 (3.9-6.1)	0.9 (0.5-1.3)
14	6.8 (5.5-8.1)	1.8 (1.1-2.5)	1.4 (0.8-2.0)	5.3 (4.2-6.4)	0.6 (0.2-0.9)	0.9 (0.4-1.3)	3.1 (2.3-4.0)	0.8 (0.4-1.3)
21	2.4 (1.6-3.2)	0.7 (0.3-1.2)	0.6 (0.2-1.0)	2.7 (1.9-3.5)	0.4 (0.1-0.7)	0.9 (0.4-1.4)	1.9 (1.2-2.6)	1.3 (0.7-1.8)
28	1.4 (0.8-2.0)	0.6 (0.2-1.0)	0.3 (0.0-0.5)	1.8 (1.1-2.5)	0.4 (0.1-0.7)	0.4 (0.1-0.8)	0.6 (0.2-1.0)	1.2 (0.6-1.7)
Days since ICU Admission, % (95% CI)								
7	-	7.1 (4.9-9.4)	12.8 (9.4-16.1)	29.3 (23.8-34.8)	0.8 (0.0-1.6)	3.9 (1.9-5.9)	9.9 (7.3-12.4)	4.6 (2.3-6.8)
14	-	3.3 (1.9-4.7)	3.0 (1.7-4.4)	20.5 (16.2-24.8)	2.3 (0.9-3.8)	3.7 (1.8-5.6)	6.1 (4.2-8)	3.8 (1.9-5.8)
21	-	1.3 (0.5-2.1)	1.1 (0.3-1.8)	10.0 (7.0-13.1)	1.6 (0.4-2.8)	3.5 (1.7-5.3)	3.6 (2.2-5.0)	5.2 (2.9-7.5)
28	-	0.9 (0.3-1.6)	0.5 (0.0-1.0)	6.5 (4.1-9.0)	1.5 (0.3-2.7)	1.7 (0.5-2.9)	1.0 (0.3-1.7)	4.6 (2.6-6.7)
Days since NIV, % (95% CI)								
7	-	8.9 (6.3-11.5)	16.1 (12.2-19.9)	23.1 (18.4-27.8)	0.6 (0.0-1.3)	2.9 (1.4-4.3)	11.7 (8.9-14.5)	2.9 (1.4-4.4)
14	-	4.1 (2.4-5.8)	3.8 (2.1-5.5)	17.2 (13.5-20.9)	1.9 (0.7-3.1)	2.9 (1.4-4.4)	7.5 (5.3-9.6)	2.8 (1.3-4.2)
21	-	1.6 (0.6-2.6)	1.3 (0.4-2.2)	8.5 (5.9-11.2)	1.3 (0.3-2.4)	2.9 (1.4-4.4)	4.4 (2.7-6.1)	4.1 (2.3-5.9)
28	-	1.2 (0.4-2.0)	0.6 (0.0-1.2)	5.6 (3.5-7.7)	1.3 (0.3-2.3)	1.4 (0.4-2.4)	1.3 (0.4-2.1)	3.8 (2.1-5.5)
Days since Intubation, % (95% CI)								
7	-	-		56.1 (48.0-64.1)	1.6 (0.0-3.3)	8.6 (4.5-12.6)	-	11.7 (6.3-17.1)
14	-	-		35.1 (28.2-42.0)	4.3 (1.7-7.0)	7.2 (3.7-10.8)	-	8.6 (4.4-12.7)
21	-	-		16.6 (11.6-21.6)	2.7 (0.7-4.8)	6 (2.9-9.0)	-	10.1 (5.9-14.3)
28	-	-		10.8 (6.7-14.8)	2.5 (0.6-4.5)	2.9 (0.9-5.0)	-	8.5 (4.8-12.2)

Footnote: All estimates are from multistate models.
Abbreviations: ICU, intensive care unit; NIV, noninvasive ventilation IMV, invasive mechanical ventilation; Hx, history.

Web Table 3 (continued). Multistate Estimates of the Proportion of Patients in Each Care State at a Given Time Point (*n* = 1,577)

	Discharged	Discharged – Hx of ICU	Discharged – Hx of IMV	Died	Died – Hx of ICU	Died – Hx of IMV
Days since Hospital Admission, % (95% CI)						
7	45.0 (42.5-47.6)	6.2 (5.0-7.4)	0.4 (0.1-0.6)	2.1 (1.4-2.9)	2.2 (1.5-2.9)	1.4 (0.8-1.9)
14	56.1 (53.6-58.7)	11.8 (10.2-13.4)	1.5 (1.0-2.1)	3.4 (2.5-4.3)	3.7 (2.7-4.6)	2.8 (2.0-3.6)
21	59.6 (57.1-62.2)	14.2 (12.5-16.0)	3 (2.2-3.8)	4.0 (3.0-5.0)	4.3 (3.3-5.3)	4.0 (3.0-4.9)
28	60.6 (58.0-63.1)	15.9 (14.0-17.7)	4.3 (3.3-5.3)	4.0 (3.0-5.0)	4.5 (3.4-5.5)	4.2 (3.2-5.2)
Days since ICU Admission, % (95% CI)						
7	-	15.6 (11.1-20.1)	1.8 (0.4-3.2)	-	7.5 (4.7-10.2)	6.8 (4.0-9.7)
14	-	26.7 (21.4-32.1)	7.2 (4.4-10.0)	-	10.8 (7.6-13.9)	12.5 (8.7-16.3)
21	-	31.4 (25.7-37.1)	13.2 (9.4-17.0)	-	12.0 (8.7-15.3)	17.2 (12.8-21.5)
28	-	34.5 (28.4-40.5)	18.4 (14.0-22.8)	-	12.3 (8.9-15.6)	18.0 (13.5-22.4)
Days since NIV, % (95% CI)						
7	-	19.0 (13.6-24.5)	1.1 (0.2-2.0)	-	9.3 (6.1-12.6)	4.4 (2.4-6.3)
14	-	32.6 (26.9-38.4)	5.0 (3.0-7.0)	-	13.4 (9.8-17.1)	8.9 (6.0-11.8)
21	-	38.3 (32.5-44.2)	9.7 (6.8-12.7)	-	15.0 (11.2-18.8)	12.8 (9.3-16.2)
28	-	42.2 (36.2-48.1)	14.0 (10.4-17.5)	-	15.3 (11.5-19.2)	13.4 (9.9-17.0)
Days since Intubation, % (95% CI)						
7	-	-	4.6 (1.0-8.1)	-	-	17.4 (11.1-23.8)
14	-	-	16.7 (10.9-22.5)	-	-	28.0 (21.1-35)
21	-	-	28.3 (21.6-35.1)	-	-	36.3 (29.1-43.4)
28	-	-	37.7 (30.6-44.7)	-	-	37.6 (30.4-44.7)

Footnote: All estimates are from multistate models.
Abbreviations: ICU, intensive care unit; NIV, noninvasive ventilation IMV, invasive mechanical ventilation; Hx, history.

Web Table 4. Length of Stay Estimates Based on Multistate Analyses (*n* = 1,577)

	**Overall**		**March 15 – May 3**	**May 4 – July 25**
Overall	5.7 (2.9-11.9)		6.9 (19.9-3.2)	5.0 (2.5-9.8)
Overall, no ICU	4.2 (2.1-7.4)		4.6 (2.4-8.8)	3.7 (2.0-6.3)
Overall, ICU no vent	8.1 (4.3-15.4)		8.5 (5.2-18.9)	7.6 (3.9-12.9)
Overall, ICU NIV	14.1 (7.3-25.8)		16.6 (8.9-28.0)	11.1 (6.8-22.1)
Overall, ICU Vent	19.1 (10.1-30.7)		21.4 (10.4-38.2)	15.9 (9.9-27.0
ICU, Overall	5.1 (2.0-11.1)		5.2 (2.0-11.8)	4.5 (1.8-10.0)
ICU, No Vent	1.9 (1.1-3.2)		1.9 (1.1-3.0)	1.9 (1.2-3.2)
ICU, NIV	4.5 (2.0-9.2)		4.5 (2.0-9.5)	5.1 (2.1-9.1)
ICU, IMV	10.3 (4.6-20.1)		10.5 (4.6-21.0	9.8 (5.1-17.3)
NIV, Overall	1.6 (0.5-3.9)		1.5 (0.4-4.0)	1.6 (0.5-3.8)
NIV, No Vent	2.1 (0.5-4.3)		1.9 (0.4-4.4)	2.2 (0.6-4.1)
NIV, IMV	1.2 (0.4-2.4)		1.1 (0.3-2.4)	1.4 (0.5-2.5)
IMV, Overall	7.2 (2.9-14.2)		9.0 (3.3-14.4)	5.6 (2.0-13.8)
IMV, No Death	9.5 (3.0-17.0)		11.0 (4.8-19.0)	6.9 (3.0-14.7)
IMV, Died	5.3 (2.3-11.3)		6.7 (3.0-11.5)	4.8 (1.4-11.3)

Abbreviations: ICU, intensive care unit; NIV, noninvasive ventilation; IMV, invasive mechanical ventilation; CI, confidence interval.

Web Table 5. Multistate Estimates Stratified by Time Period (*n* = 1,577)

	Inpatient Floor	ICU	NIV	IMV	NIV – Hx of IMV	ICU – Hx of IMV	Inpatient Floor – Hx of ICU	Inpatient Floor – Hx of IMV
Days since Hospital Admission, % (95% CI)								
March 15 – May 3								
14	8.3 (6.3-10.2)	2.2 (1.1-3.2)	1.4 (0.6-2.3)	6.7 (4.9-8.4)	0.8 (0.2-1.4)	0.9 (0.3-1.5)	3.6 (2.4-4.9)	0.9 (0.3-1.5)
28	2.2 (1.2-3.3)	1.0 (0.3-1.6)	0.1 (0.0-0.3)	1.9 (1.0-2.8)	0.6 (0.1-1.1)	0.4 (0.0-0.9)	0.9 (0.3-1.5)	1.9 (1.0-2.8)
May 4 – July 25								
14	5.1 (3.4-6.8)	1.5 (0.6-2.4)	1.3 (0.5-2.1)	3.9 (2.6-5.3)	0.4 (0.0-0.7)	0.8 (0.3-1.4)	2.4 (1.3-3.5)	0.8 (0.2-1.3)
28	0.3 (0.0-0.7)	0.0 (0.0-0.0)	0.6 (0.0-1.3)	1.8 (0.9-2.8)	0.2 (0.0-0.4)	0.3 (0.0-0.8)	0.2 (0.0-0.4)	0.3 (0.0-0.6)
Days since ICU Admission, % (95% CI)								
March 15 – May 3								
14	-	3.1 (1.2-5.0)	2.4 (0.8-4.1)	23.5 (16.5-30.4)	2.8 (0.6-5.0)	3.4 (0.9-5.9)	6.2 (3.3-9.2)	3.9 (1.0-6.9)
28	-	1.1 (0.2-1.9)	0.2 (0.0-0.4)	6.3 (3.1-9.5)	2.0 (0.3-3.7)	1.6 (0.1-3.1)	1.3 (0.3-2.2)	6.9 (3.4-10.4)
May 4 – July 25								
14	-	3.5 (1.4-5.6)	3.4 (1.2-5.6)	17.3 (11.7-23.0)	1.8 (0-3.6.0)	4.2 (1.3-7.1)	5.4 (2.8-8.0)	4.1 (1.2-7.0)
28	-	0.0 (0.0-0.0)	1.5 (0.0-2.9)	7.6 (3.7-11.5)	0.8 (0.0-1.9)	1.5 (0.0-3.2)	0.4 (0.0-0.9)	1.3 (0.0-2.9)
Days since NIV, % (95% CI)								
March 15 – May 3								
14	-	4.0 (1.8-6.1)	3.1 (1.2-5.0)	19.7 (14.1-25.2)	2.3 (0.5-4.1)	2.7 (0.8-4.6)	7.9 (4.8-11.0)	2.8 (0.7-4.8)
28	-	1.4 (0.3-2.4)	0.2 (0.0-0.5)	5.4 (2.7-8.1)	1.7 (0.2-3.1)	1.3 (0.1-2.5)	1.6 (0.4-2.8)	5.6 (2.9-8.4)
May 4 – July 25								
14	-	4.2 (1.7-6.7)	4.1 (1.6-6.7)	15.0 (9.9-20.1)	1.5 (0.0-3.0)	3.4 (1.0-5.7)	6.3 (3.3-9.3)	3.2 (0.9-5.5)
28	-	0.0 (0.0-0.0)	1.8 (0.1-3.5)	6.7 (3.2-10.2)	0.7 (0.0-1.6)	1.3 (0.0-2.8)	0.4 (0.0-1.1)	1.1 (0.0-2.4)
Days since Intubation, % (95% CI)								
March 15 – May 3								
14	-	-		37.2 (27.7-46.7)	4.6 (1.2-8.1)	5.9 (1.8-10.0)	-	8.2 (2.6-13.8)
28	-	-		9.7 (4.9-14.5)	3.1 (0.5-5.7)	2.6 (0.2-4.9)	-	11.5 (6.2-16.8)
May 4 – July 25								
14	-	-		31.1 (21.1-41.1)	3.7 (0.0-7.5)	8.8 (3.0-14.7)	-	9.4 (3.1-15.7)
28	-	-		13.0 (6.2-19.7)	1.3 (0.0-3.2)	2.6 (0.0-5.6)	-	2.7 (0.0-5.9)

Web Table 5 (continued). Multistate Estimates of the Proportion of Patients in Each Care State at a Given Time Point (*n* = 1,577)

	Discharged	Discharged – Hx of ICU	Discharged – Hx of IMV	Died	Died – Hx of ICU	Died – Hx of IMV
Days since Hospital Admission, % (95% CI)						
March 15 – May 3						
14	50.9 (47.3-54.5)	9.3 (7.3-11.4)	1.3 (0.5-2.0)	4.9 (3.3-6.4)	5.3 (3.8-6.9)	3.6 (2.3-4.8)
28	55.4 (51.8-59.0)	13.7 (11.4-16.1)	4.2 (2.9-5.6)	5.8 (4.1-7.4)	6.5 (4.8-8.2)	5.3 (3.8-6.9)
May 4 – July 25						
14	61.8 (58.2-65.5)	14.4 (11.8-16.9)	1.8 (0.9-2.6)	1.7 (0.8-2.7)	2.0 (1.0-2.9)	2.0 (1.1-3.0)
28	66.3 (62.7-69.8)	18.3 (15.5-21.1)	4.5 (3.1-5.9)	1.9 (0.9-2.9)	2.3 (1.2-3.3)	3.0 (1.8-4.1)
Days since ICU Admission, % (95% CI)						
March 15 – May 3						
14	-	19.7 (11.7-27.8)	6.0 (1.9-10.0)	-	13.8 (7.9-19.8)	15.1 (8.4-21.9)
28	-	26.7 (17.0-36.4)	17 (10.3-23.7)	-	15.6 (9.2-22.0)	21.4 (13.5-29.4)
May 4 – July 25						
14	-	33.9 (26.3-41.4)	9.5 (5.0-14.0)	-	6.3 (3.0-9.7)	10.6 (5.8-15.4)
28	-	42.7 (34.2-51.3)	22.2 (15.5-29.0)	-	7.1 (3.6-10.6)	15.0 (9.4-20.6)
Days since NIV, % (95% CI)						
March 15 – May 3						
14	-	25.1 (17.1-33.2)	3.9 (1.3-6.6)	-	17.7 (11.7-23.7)	10.8 (6.0-15.7)
28	-	34 (25.4-42.6)	12.8 (7.9-17.7)	-	20.0 (13.6-26.3)	16.1 (10.3-21.9)
May 4 – July 25						
14	-	39.4 (31.0-47.7)	7.2 (3.5-10.8)	-	7.8 (3.7-11.9)	8.0 (4.1-11.9)
28	-	49.9 (41.0-58.8)	17.7 (11.7-23.6)	-	8.7 (4.4-12.9)	11.7 (7.0-16.5)
Days since Intubation, % (95% CI)						
March 15 – May 3						
14	-	-	13.3 (5.5-21.2)	-	-	30.7 (21.0-40.4)
28	-	-	32.4 (22.9-41.8)	-	-	40.8 (30.9-50.6)
May 4 – July 25						
14	-	-	22.4 (13.2-31.6)	-	-	24.5 (14.9-34.2)
28	-	-	47.6 (37.0-58.2)	-	-	32.8 (22.6-43.0)

Footnote: All estimates are from multistate models.

Abbreviations: ICU, intensive care unit; NIV, noninvasive ventilation IMV, invasive mechanical ventilation; Hx, history.

Web Table 6. Stratified Estimates of the Cumulative Incidence of ICU Admission, NIV, Intubation, and Death by 28 Days

	ICU, % (95% CI)	NIV, % (95% CI)	Intubation, % (95% CI)	Death, % (95% CI)
Overall	36.3 (33.9-38.7)	22.0 (19.9-24.1)	13.9 (12.2-15.6)	13.1 (11.4-14.9)
Female	30.0 (26.8-33.3)	18.7 (16.0-21.5)	10.5 (8.4-12.7)	11.4 (9.2-13.8)
Male	42.6 (39.1-46.1)	25.3 (22.3-28.4)	17.3 (14.7-20.1)	14.8 (12.4-17.5)
<50 years old	26.8 (22.4-31.3)	13.6 (10.3-17.2)	8.7 (6.2-11.9)	1.7 (0.7-3.4)
50-70 years old	39.7 (35.9-43.5)	25.5 (22.2-29.0)	16.9 (14.1-20.0)	8.6 (6.5-11.0)
>70 years old	39.1 (34.9-43.3)	23.8 (20.2-27.5)	13.8 (10.9-16.9)	27.1 (23.3-31.1)
Black	35.2 (32.1-38.3)	20.6 (18.0-23.3)	13.7 (11.5-16.0)	12.0 (9.9-14.2)
White	36.9 (32.9-41.0)	24.1 (20.6-27.8)	13.3 (10.6-16.3)	14.8 (11.9-17.9)
Community	34.6 (31.9-37.3)	20.9 (18.7-23.3)	13.6 (11.7-15.7)	7.4 (6.0-9.0)
Long Term Care Facility	42.0 (36.9-47.1)	25.4 (21.0-30.0)	14.6 (11.2-18.5)	31.4 (26.6-36.3)
No Diabetes	34.9 (31.8-38.1)	20.6 (18.0-23.4)	13.6 (11.4-15.9)	11.7 (9.6-14.0)
Diabetes	38.1 (34.4-41.8)	23.8 (20.6-27.1)	14.2 (11.7-17.0)	14.9 (12.3-17.8)
No Hypertension	35.0 (30.2-39.8)	18.5 (14.7-22.6)	13.3 (10.1-16.9)	6.3 (4.2-9.2)
Hypertension	36.7 (33.9-39.5)	23.1 (20.7-25.6)	14.0 (12.1-16.1)	15.3 (13.2-17.4)
No Chronic Kidney Disease	35.5 (32.7-38.4)	21.6 (19.1-24.1)	13.2 (11.2-15.3)	10.5 (8.7-12.5)
Chronic Kidney Disease	37.9 (33.6-42.3)	22.9 (19.2-26.8)	15.4 (12.3-18.8)	18.8 (15.4-22.5)
No Cardiac Disease	34.6 (31.4-37.7)	20.0 (17.4-22.7)	13.4 (11.3-15.8)	9.2 (7.3-11.2)
Cardiac Disease	38.6 (34.9-42.3)	24.6 (21.4-28.0)	14.4 (11.8-17.2)	18.2 (15.3-21.3)
No Pulmonary Disease	35.5 (32.6-38.4)	21.3 (18.9-23.9)	14.3 (12.3-16.5)	12.9 (10.9-15.0)
Pulmonary Disease	38.2 (33.8-42.5)	23.5 (19.7-27.4)	12.7 (9.9-16.0)	13.6 (10.6-17.0)
No Tobacco Abuse	36.2 (33.1-39.3)	22.5 (19.8-25.2)	15.1 (12.8-17.5)	13.5 (11.4-15.9)
Tobacco Abuse	36.5 (32.7-40.3)	21.3 (18.1-24.6)	12.0 (9.6-14.8)	12.4 (9.9-15.2)
No Obesity	37.1 (33.5-40.6)	21.7 (18.7-24.8)	15.6 (13.0-18.3)	14.3 (11.8-17.0)
Obesity	35.6 (32.4-38.9)	22.3 (19.5-25.2)	12.4 (10.2-14.8)	12.0 (9.9-14.4)
March 15 - May 3	37.9 (34.5-41.3)	22.4 (19.5-25.4)	15.2 (12.8-17.8)	17.8 (15.2-20.6)
May 4 – July 25	34.5 (31.1-37.9)	21.5 (18.6-24.5)	12.4 (10.1-14.9)	7.9 (6.0-10.0)

Abbreviations: ICU, intensive care unit; NIV, noninvasive ventilation; CI, confidence interval.

Web Table 7. Adjusted Age-Stratified Estimates of ICU Admission, Noninvasive Ventilation, Intubation, and Death by Time Period (*n* = 1,577)

	**March 15 – May 3**	**May 4 – July 25**
Age <50 years, % (95% CI)		
ICU	24.9 (17.2-32.7)	28.8 (22.2-35.4)
NIV	9.3 (5.1-13.5)	17.6 (11.7-23.5)
Intubation	8.0 (3.9-12.2)	8.4 (4.7-12.2)
Died	0.9 (0.0-2.0)	2.1 (0.0-4.2)
Age 50-70 years, % (95% CI)		
ICU	35.4 (29.6-41.3)	46.1 (37.4-54.9)
NIV	20.9 (16.4-25.3)	33.1 (25.4-40.9)
Intubation	15.7 (11.8-19.6)	18.0 (12.6-23.4)
Died	10.8 (7.6-14.1)	5.6 (2.4-8.8)
Age >70 years, % (95% CI)		
ICU	36.0 (29.5-42.6)	42.5 (33.1-52.0)
NIV	23.2 (18.0-28.5)	23.5 (16.6-30.5)
Intubation	13.5 (9.5-17.6)	13.6 (8.3-18.9)
Died	31.5 (25.3-37.6)	20.1 (13.6-26.6)

Marginal estimates were obtained from Poisson models adjusted for sex, race, patient comorbidities, and whether patients came from a long-term care facility with a time offset.
Abbreviations: ICU, intensive care unit; NIV, noninvasive ventilation; CI, confidence interval.

Web Figure 1

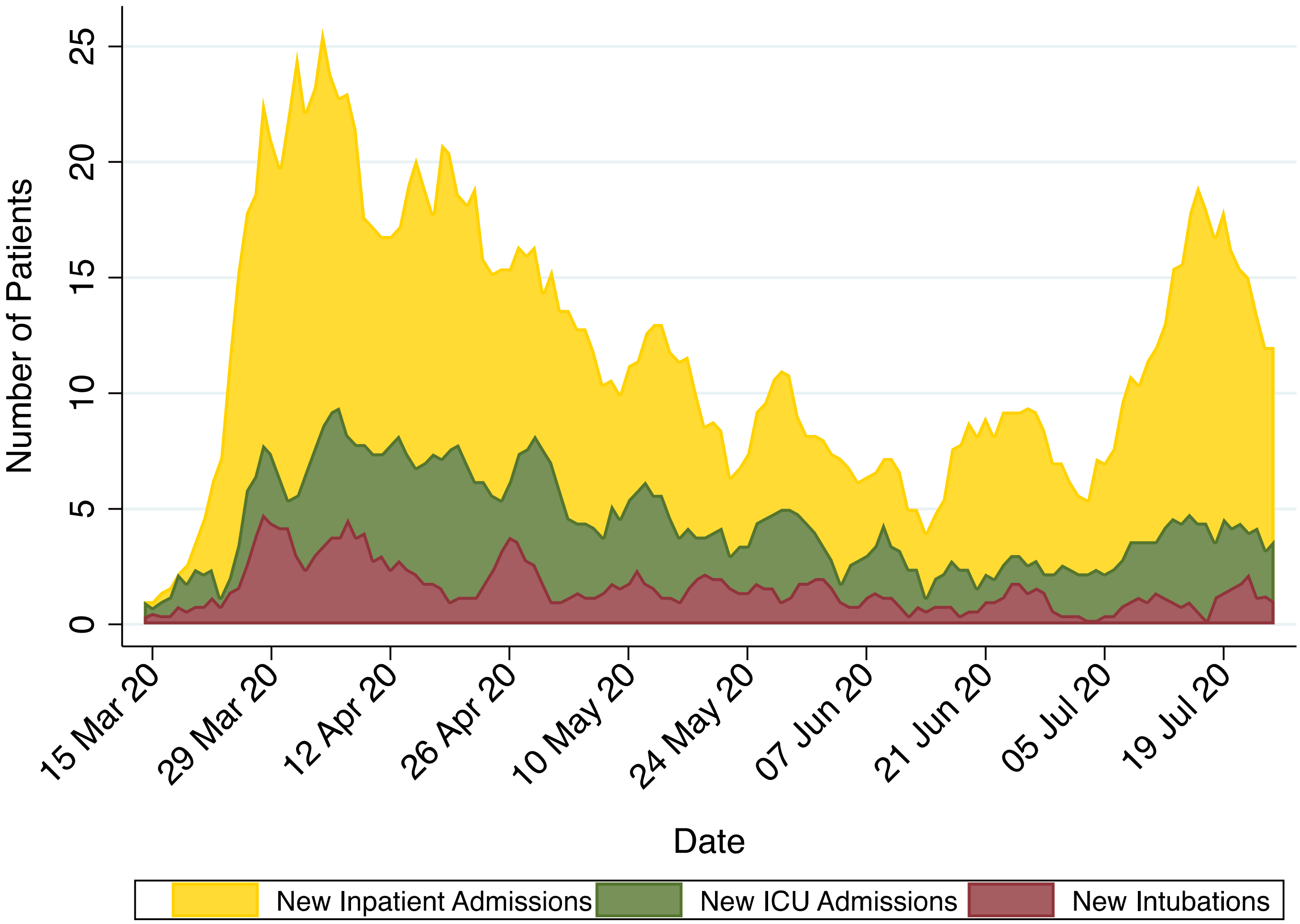

Web Figure 1. Daily Number of New Inpatient Admissions, ICU admissions, and Intubations. Daily trends are based on five-day moving averages.
